# Host-Defense-Peptide-Mimicking β-Peptide Polymer Acting as a Dual-Modal Antibacterial Agent by Interfering Quorum Sensing and Killing Individual Bacteria Simultaneously

**DOI:** 10.34133/research.0051

**Published:** 2023-03-14

**Authors:** Wanlin Li, Ximian Xiao, Yuchen Qi, Xiuhui Lin, Huiqun Hu, Minqi Shi, Min Zhou, Weinan Jiang, Longqiang Liu, Kang Chen, Kai Wang, Runhui Liu, Min Zhou

**Affiliations:** ^1^ Department of Respiratory and Critical Care Medicine, the Fourth Affiliated Hospital, Zhejiang University School of Medicine, Yiwu 223300, China.; ^2^ University-University of Edinburgh Institute (ZJU-UoE Institute), Zhejiang University School of Medicine, Zhejiang University, Haining 314400, China.; ^3^ State Key Laboratory of Bioreactor Engineering, Key Laboratory for Ultrafine Materials of Ministry of Education, Frontiers Science Center for Materiobiology and Dynamic Chemistry, Research Center for Biomedical Materials of Ministry of Education, School of Materials Science and Engineering, East China University of Science and Technology, Shanghai 200237, China.; ^4^ Institute of Translational Medicine, Zhejiang University, Hangzhou 310029, China.; ^5^ Department of Infectious Diseases, The Second Affiliated Hospital, Zhejiang University School of Medicine, Hangzhou 310058, China; ^6^ State Key Laboratory of Modern Optical Instrumentations, Zhejiang University, Hangzhou 310058, China.

## Abstract

Host defense peptides (HDPs) are one of the potentially promising agents for infection diseases due to their broad spectrum and low resistance rate, but their clinical applications are limited by proteolytic instability, high-cost, and complicated synthesis process. Here, we report a host-defense-peptide-mimicking β-peptide polymer that resists proteolysis to have enhanced the activity under physiological conditions, excellent antimicrobial efficiency even at high density of bacteria, and low cost for preparation. The β-peptide polymer demonstrated quorum sensing (QS) interference and bactericidal effect against both bacterial communities and individual bacterium to simultaneously block bacterial communication and disrupt bacterial membranes. The hierarchical QS network was suppressed, and main QS signaling systems showed considerably down-regulated gene expression, resulting in excellent biofilm eradication and virulence reduction effects. The dual-modal antibacterial ability possessed excellent therapeutic effects in *Pseudomonas aeruginosa* pneumonia, which could inhibit biofilm formation and exhibit better antibacterial and anti-inflammatory efficiency than clinically used antibiotics, levofloxacin. Furthermore, the β-peptide polymer also showed excellent therapeutic effect *Escherichia coli* pyogenic liver abscess. Together, we believed that the β-peptide polymer had a feasible clinical potential to treat bacterial infection diseases.

## Introduction

Bacterial infection diseases, such as bacterial pneumonia and pyogenic liver abscess, are severe threats to human health and contribute to predominant morbidity and mortality historically [1–4]. In the last few decades, the decreased efficiency of conventional antibiotics due to the emergence of multidrug-resistant bacteria makes infections a more severe challenge that attracts global concern [5–7]. Therefore, new agents against infections are needed urgently. In addition to changes in the resistance mechanism of individual bacteria, the emergence of drug resistance is also a social behavior of bacteria [8–10]. While the bacterial population density reaches a certain threshold, the bacterial communication network named quorum sensing (QS) is activated [11,12]. Bacteria release QS signal molecules to regulate virulence and extracellular polymeric substance composition to enhance invasiveness themselves and resist the clearance of the immune system [13,14]. The secreted extracellular polymeric substances envelop viable bacteria tightly, making antibiotics difficult to penetrate and kill the bacteria, which is a vital source of drug resistance [15–17]. Now, increasing researches have proven the close relationship between QS and clinical bacterial diseases [18–21]. The subsequently formed biofilms make bacteria easier to exposure to sublethal concentrations [22–25], thereby developing new resistance mechanisms. Therefore, QS suppression and biofilm inhibition are essential parts to combat bacterial infection diseases [26].

**Fig. 1.**
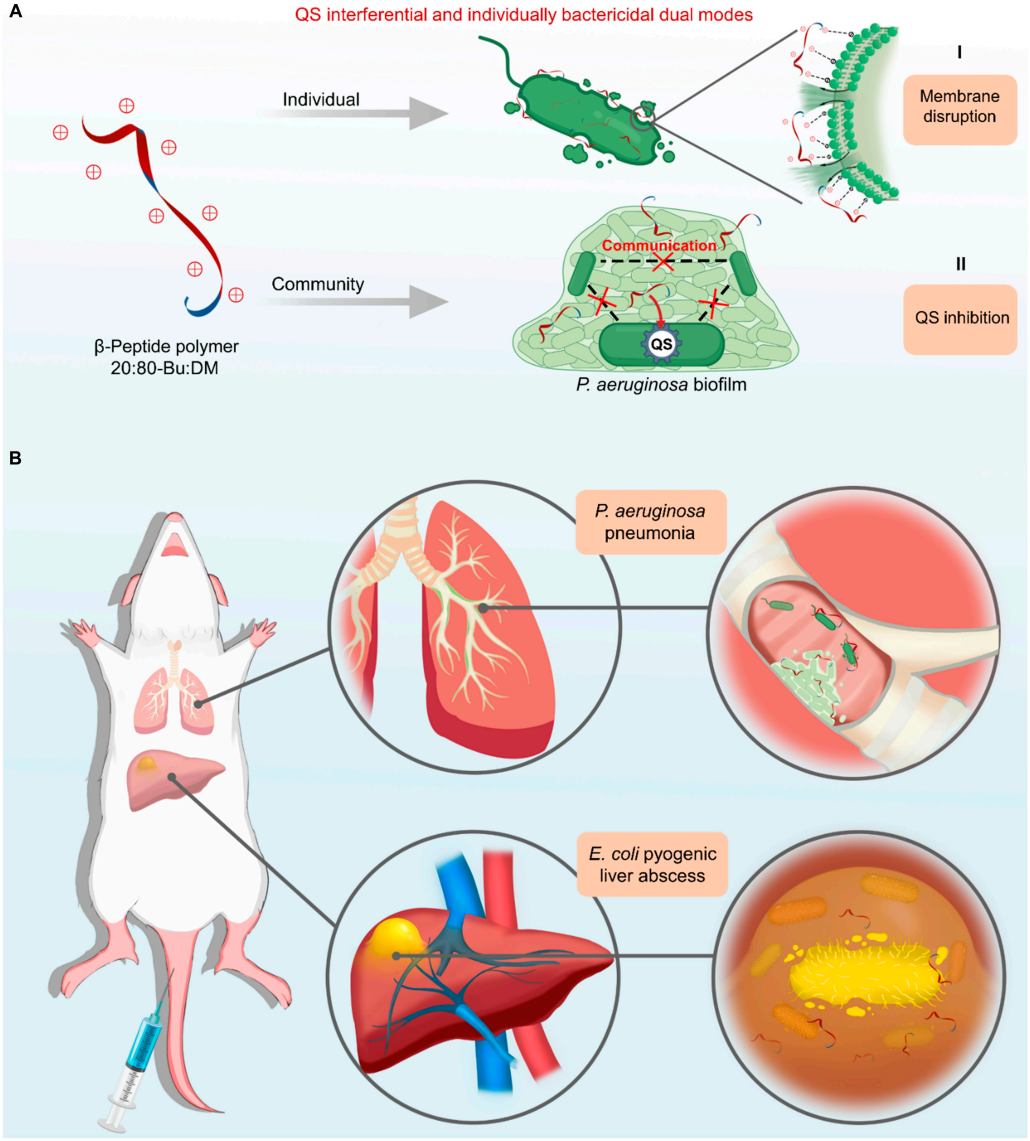
20:80-Bu:DM inhibits bacteria and cures *P. aeruginosa* pneumonia and ECPLA by QS interferential and individually bactericidal dual modes. (A) 20:80-Bu:DM inhibits individual bacterium using a membrane-targeted antimicrobial mechanism, inhibits biofilm, and reduces bacteria virulence by its anti-QS activities. (B) 20:80-Bu:DM is effective against *P. aeruginosa* pneumonia and ECPLA.

Host defense peptides (HDPs) are one of the most promising strategies to supplement and enhance current therapies [27–30] due to their broad-spectrum antibacterial ability and lower risk of inducing drug resistance [31,32]. Their small size and amphipathicity derived from the amino acid scaffolds mean that they are excellent candidates for new drug development to act on the blood circulatory system and heal visceral infectious diseases [29]. Despite these advantages, the clinical use of HDPs is still hindered by some shortcomings, including proteolytic instability and high cost. To address the prominent limitations of HDP and find promising antibacterial agents, scholars turned attention to HDP-mimicking peptides and polymers [33–41]. We recently developed an HDP-mimicking β-peptide polymer 20:80-Bu:DM (where Bu is a hydrophobic subunit and DM is a hydrophilic/cationic subunit) to solve this problem and provide another way of thinking for clinical application. Unnatural β-amino acids were incorporated to construct the peptide scaffold, helping 20:80-Bu:DM to resist proteolysis. The broad-spectrum and instant antimicrobial activity of the β-peptide polymer have been studied in our previous work [42,43]. Moreover, 20:80-Bu:DM shows a great antibacterial effect on multidrug-resistant bacteria strains without new drug resistance generation [43]. We further explored the antimicrobial mode and translational potential of 20:80-Bu:DM in treating infection diseases. The membrane-targeting antimicrobial capabilities of the 20:80-Bu:DM conduct the effective and rapid lysis of individual bacterium. In addition, for biofilms, a more common form of the bacterial community and a more severe challenge for health care, 20:80-Bu:DM could also penetrate and kill internal viable bacteria. Furthermore, we analyzed the inhibition effect of 20:80-Bu:DM on the QS system of *Pseudomonas aeruginosa* and found that the intercellular communication system of bacteria was blocked by 20:80-Bu:DM, resulting in the reduced secretion of virulence factors and decreased pathogenicity (Fig. 1). The dual-modal antibacterial effects were validated to be effective in the *P. aeruginosa* pneumonia model to eradicate viable bacteria in organs and inhibit the formation of biofilms. In addition, for the *Escherichia coli* pyogenic liver abscess (ECPLA), the 20:80-Bu:DM was also found to have a great effect.

## Results and Discussion

### Synthesis and characterizations of the 20:80-Bu:DM

**Fig. 2.**
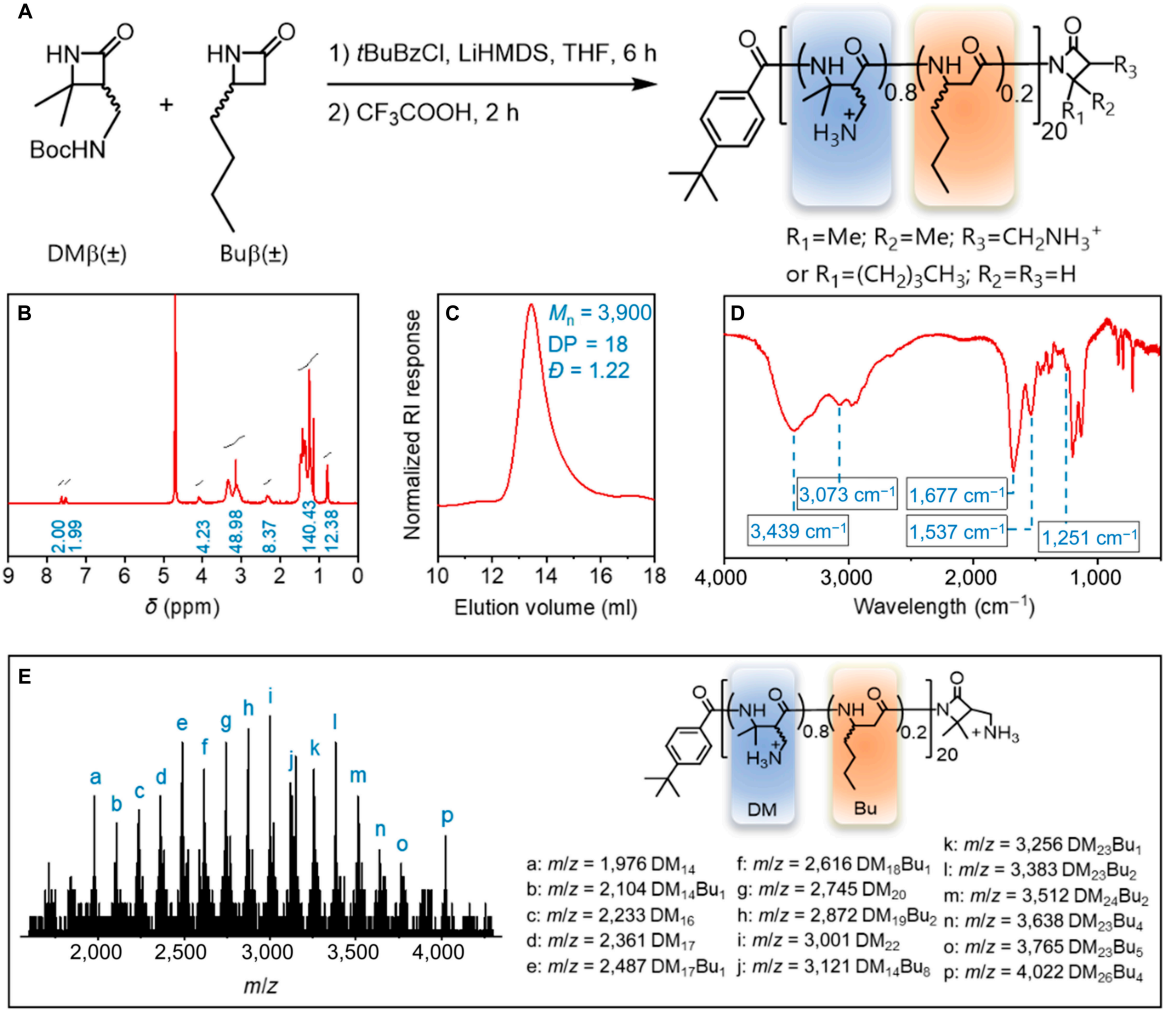
Synthesis and characterizations of the 20:80-Bu:DM. (A) Synthesis of β-peptide polymer 20:80-Bu:DM. (B) ^1^H NMR characterization of deprotected 20:80-Bu:DM. (C) GPC traces of side-chain N-*tert*-butyloxycarbony (NHBoc) protected 20:80-Bu:DM. (D) Fourier transform infrared spectroscopy characterization of deprotected 20:80-Bu:DM. (E) Matrix-assisted laser desorption/ionization-time-of-flight mass spectrometry characterization of deprotected 20:80-Bu:DM, the analysis of peaks, and the corresponding chemical structures ([M + Na]^+^).

To simulate the amphiphilic structure and positive charge of HDP, we successfully prepared a library of β-peptide polymer in the previously reported literature [43]. The optimized 20:80-Bu:DM showed excellent broad-spectrum antibacterial activities against a variety of Gram-positive and Gram-negative bacteria, including clinical multidrug-resistant strains [42,43]. The optimal β-peptide polymer 20:80-Bu:DM was synthesized via an anion ring-opening polymerization of β-lactam monomers DM and Bu, followed by treatment with trifluoroacetic acid to remove the protecting group (Fig. 2A). The number–average molecular weight (*M*_n_) was confirmed using ^1^H nuclear magnetic resonance (NMR) and gel permeation chromatography (GPC) characterizations for deprotected and protected 20:80-Bu:DM, respectively (Fig. 2B and C). The copolymer ratio of 20:80 hydrophobic:charged units was obtained by the appropriate feed ratio of the monomers, and the composition of 20:80-Bu:DM was confirmed by ^1^H NMR. Fourier transform infrared spectroscopy showed the amide characteristic bands of 20:80-Bu:DM (Fig. 2D). Figure 2E displays the matrix-assisted laser desorption/ionization-time-of-flight mass spectrometry of 20:80-Bu:DM, and the molecular weight intervals agreed with the mass of DM or Bu.

### 20:80-Bu:DM possesses superior antimicrobial efficiency and stability

**Fig. 3.**
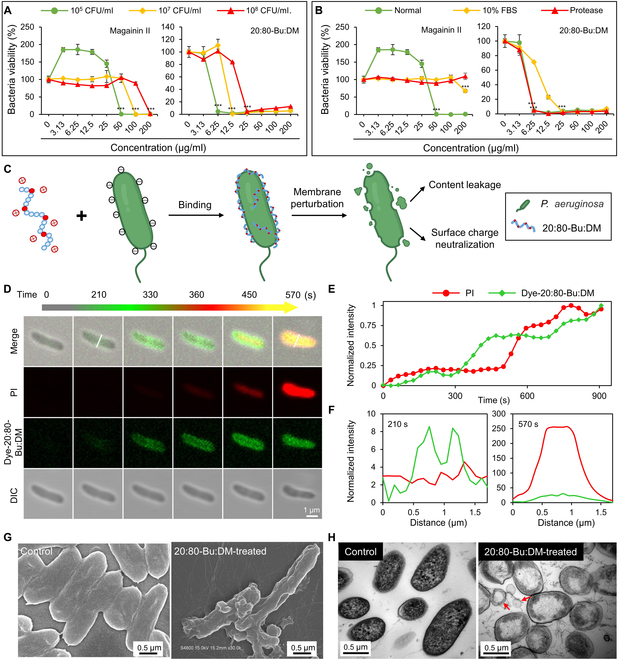
20:80-Bu:DM has higher membrane-targeted antimicrobial efficiency and stability compared with the commercial peptide. (A) Antibacterial activity of magainin II (left) and 20:80-Bu:DM (right) against PAO1 with different bacterial concentrations (*n* = 3). (B) Effects of the simulated physiological conditions and trypsin treatment on the antibacterial activity of magainin II (left) and 20:80-Bu:DM (right) (*n* = 3). (C) Illustration of the membrane-targeted disruption and cell lysis caused by peptides. (D) Time-lapse fluorescent confocal imaging of bacteria, green fluorescence indicated the dye-20:80-Bu:DM, and red fluorescence indicated that the PI penetrated the disrupted cell membrane and combined with the internal DNA (2 × MIC). (E) Fluorescence intensity of dye-20:80-Bu:DM and PI versus time. (F) Fluorescence intensity profiles of dye-20:80-Bu:DM and PI across PAO1 at 210 s (left) and 570 s (right). (G and H) SEM and TEM images of the peptides and treated bacteria respectively. Membrane disruption is showed in the images, and the cell content leakage is observed in the TEM images as the red arrows indicated. Significance of differences was determined using the one-way ANOVA method. ***P < 0.001. DIC, differential interference contrast.

We evaluated the antimicrobial activities of the 20:80-Bu:DM against 2 strains used for infection models, and the minimum inhibitory concentration (MIC) values of the 20:80-Bu:DM were 6.25 μg/ml for *P. aeruginosa* and 25 μg/ml for *E. coli* (Fig. S1). To explain the advantages of the 20:80-Bu:DM applied in practical cases that have higher bacterial concentrations than the standard antimicrobial tests, we raised the bacterial concentrations to compare the antimicrobial efficiency of 20:80-Bu:DM and commercially used natural peptide magainin II (Fig. 3A). When the bacterial concentration was raised to 10^7^ colony-forming units (CFU)/ml, reaching 100 times the bacterial concentration used in the standard MIC test, 20:80-Bu:DM and magainin II could inhibit the growth of *P. aeruginosa* at 12.5 and 100 μg/ml, respectively. Once the bacterial concentration was raised to 10^8^ CFU/ml, 20:80-Bu:DM could inhibit the bacterial growth of *P. aeruginosa* at 25 μg/ml. As a comparison, only when the concentration of magainin II was 200 μg/ml and above, it can inhibit bacterial growth.

We further evaluated the stability of 20:80-Bu:DM at the simulated physiological conditions for its potential downstream application in vivo. As treatment with trypsin for 40 min, the MIC value of 20:80-Bu:DM remained unchanged, while magainin II lost its activity completely after the same treatment (Fig. 3B). Then, the 10% fetal bovine serum (FBS) was used to simulate the serum environment for antimicrobial activity test. The MIC of 20:80-Bu:DM slightly increased from 6.25 to 25 μg/ml, while the MIC of magainin II increased from 50 μg/ml to more than 200 μg/ml. We evaluated the serum stability of the β-peptide polymer, 20:80-Bu:DM, by incubating the polymer in FBS for 1 week. Our attempt to evaluate the stability of 20:80-Bu:DM by analyzing the possible change of GPC trace using water as the mobile phase was not achieved because of the overlapping of peaks between FBS and the β-peptide polymer (Fig. S1B). Therefore, we evaluated the stability of this β-peptide polymer in serum by measuring the polymer’s antibacterial activity after incubation with serum. We observed that the MIC of 20:80-Bu:DM slightly changed from 6.25 to 3.13 μg/ml after the polymer was treated in FBS (Fig. S1C). This result indicates that 20:80-Bu:DM had superior stability in serum and was reasonably applied for in vivo study. Our result is consistent with the report in precedent literatures that β-peptides and β-peptide polymers have excellent stability and resist proteolysis [44–46].

The cytotoxicity was evaluated with the NIH-3T3, Beas-2B, and A549 cell lines (Fig. S2A to C). The cell viability was over 80% for all the 3 cell lines at the concentration of 50 μg/ml. The live/dead double staining also showed a satisfactory survival of the cells at 25 μg/ml (Fig. S2D).

### 20:80-Bu:DM could selectively disrupt bacterial membrane

The positively charged 20:80-Bu:DM has preferential electrostatic interactions with the bacterial cells that have more negative charges on their surfaces compared with mammalian cells (Fig. 3C) [47]. The bacteria (93%) were found to have a ruptured or permeable membrane and eventually died, which was evaluated by SYTO 9/propidium iodide (PI) costaining, a commonly used method for membrane integrity analysis (Fig. S3) [48]. We further synthesized a morpholino-naphthalimide fluorophore-conjugated 20:80-Bu:DM (dye-20:80-Bu:DM; Figs. S4 to S7) for time-lapse fluorescent confocal imaging to understand the bactericidal process (Fig. 3D). The nucleic acid dye PI was served as an indicator to indicate the integrity of bacterial membranes. After *P. aeruginosa* was incubated with dye-20:80-Bu:DM and PI for about 210 s, dye-20:80-Bu:DM was enriched on the cell membrane. Until 240 s, a small amount of dye-20:80-Bu:DM crossed the cell membrane, followed by almost simultaneous PI entering into the cell membrane and emitting red fluorescence. PI had an obvious enrichment in bacteria at 570 s, indicating that the bacterial membrane had been damaged and the bacteria were killed by dye-20:80-Bu:DM. The fluorescence intensity was measured and showed a consistent changing rule (Fig. 3E and F). Membrane labeling dye FM 4-64 (red fluorescence) was used to visualize the membrane perturbation (Fig. S8). With the time increasing, the membrane outline was gradually blurred and finally disappeared at approximately 120 min. After the bacterial membrane was completely eroded at 12 h, the bacteria demonstrated unusual aggregation and morphological changes induced by neutralization of bacterial surface charge. These phenomena also could be observed in the scanning electron microscope (SEM) imaging (Fig. 3G). Meanwhile, the transmission electron microscope (TEM) images clearly showed the leakage of cell content assembly led by transmembrane pores (Fig. 3H and Fig. S9). Our antibacterial mechanism study, including aforementioned time-lapse fluorescent confocal imaging, SEM characterization, and TEM characterization, indicate that the β-peptide polymer electrostatically binds to the surface of bacterial cell wall and then penetrates the cell wall after interaction with the outer membrane using its amphiphilic structural characteristics. We hypothesized that during this process, the polymer can depolarize the inner membrane and finally destroy the bacterial cell wall and inner membrane to kill bacteria. Our antibacterial mechanism study and hypothesis are consistent with the action modes of some HDPs in precedent literatures [49,50].

### 20:80-Bu:DM demonstrates excellent biofilm elimination activity

**Fig. 4.**
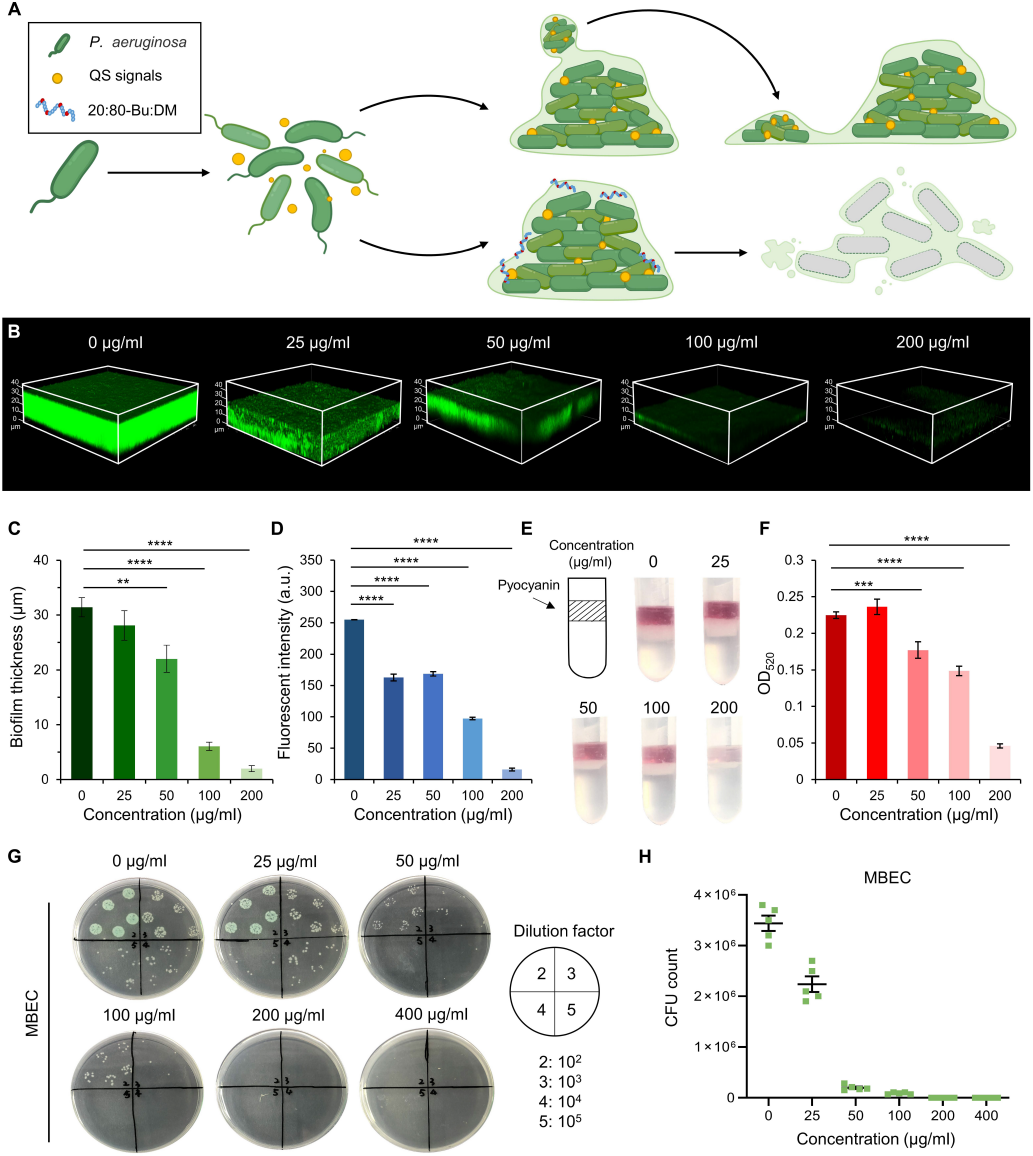
20:80-Bu:DM can eradicate *P. aeruginosa* biofilms and suppress PYO secretion. (A) A schematic diagram of the peptides disrupt biofilm to inhibit bacteria proliferation and expansion. (B) SYTO 9 stained biofilm treated with different concentrations of 20:80-Bu:DM. (C) Biofilms treated with 20:80-Bu:DM showed significantly reduced thickness (*n* = 5). (D) Bacteria density of the biofilms treated with 20:80-Bu:DM, which was quantified with the fluorescent intensity of SYTO 9, showed a significant reduction (*n* = 5). (E and F) PYO secretion of the PAO1 with different concentrations of 20:80-Bu:DM treatment. Images of the extracted PYO were taken, and the PYO content is quantified with absorbance at 520 nm (*n* = 5). (G) PAO1 biofilms were incubated with different concentrations of 20:80-Bu:DM, and the viable bacteria number in biofilms was calculated to determine the MBEC of the peptide. (H) Viable bacteria curve in biofilms after gradient concentrations of the 20:80-Bu:DM treatments (*n* = 5). Significance of differences was determined using the one-way ANOVA method. **P < 0.01, ***P < 0.001, and ****P <0.0001. a.u., arbitrary units; OD, optical density.

Many studies have demonstrated the advantages of cationic HDPs in penetrating biofilms [51–53]; thus, we believed that 20:80-Bu:DM also has the potential for antibiofilm application (Fig. 4A). To investigate the antibiofilm activity of 20:80-Bu:DM, we incubated mature *P. aeruginosa* biofilms with different concentrations of 20:80-Bu:DM and stained those with SYTO 9 for evaluation. From the three-dimensional reconstruction models of the biofilms (Fig. 4B), we analyzed the thickness (Fig. 4C) and bacteria density (indicated with the fluorescent intensity of SYTO 9) of the biofilms (Fig. 4D). 20:80-Bu:DM demonstrated a biofilm inhibitory effect with the concentration increasing. The thickness and bacteria density of biofilms showed marked reduction at the concentration of 50 μg/ml, and the biofilm was almost eradicated at the concentration of 200 μg/ml. The number of viable bacteria in biofilms was counted, and the results were shown in Fig. 4G and H. In line with the above data, bacteria were eliminated under the treatment of 20:80-Bu:DM (200 μg/ml), which was determined as the minimum biofilm eradication concentration (MBEC) value. The pyocyanin (PYO), a representative virulence factor secreted by *P. aeruginosa* during biofilm formation, was extracted and quantified (Fig. 4E and F). The PYO secretion of *P. aeruginosa* exhibited a significant decrease when the concentration of 20:80-Bu:DM reached 50 μg/ml, and it was hard to detect the PYO when the concentration of 20:80-Bu:DM reached 200 μg/ml, demonstrating the effective antivirulence and antibiofilm effect of 20:80-Bu:DM.

### 20:80-Bu:DM suppresses QS of bacteria

**Fig. 5.**
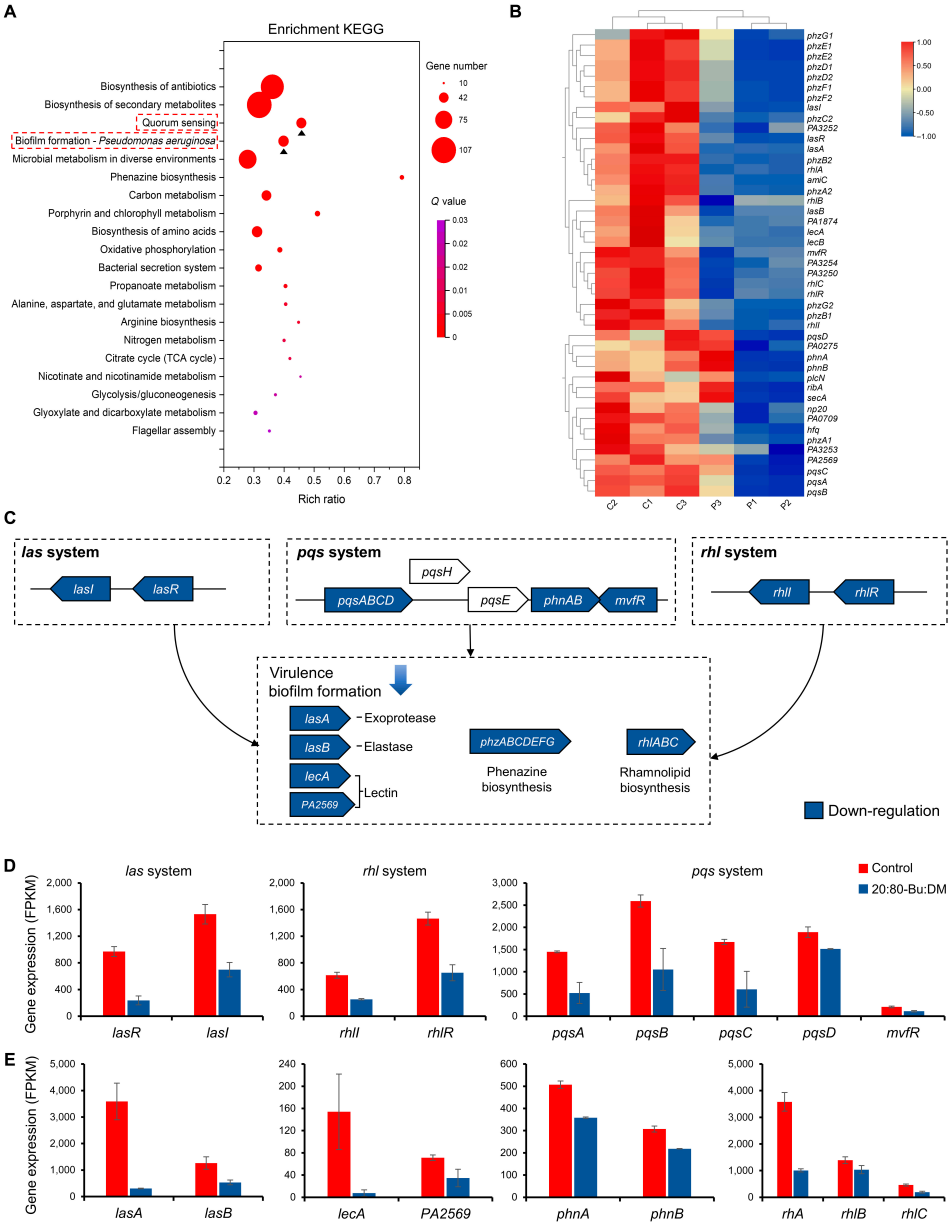
20:80-Bu:DM inhibits the QS of *P. aeruginosa* and reduces the bacterial virulence. (A) The bubble chart of the KEGG pathway enrichment analysis, demonstrating the top 20 enrichment entries including the QS and biofilm formation pathway that are marked with a triangle and red box. (B) The clustering heatmap of QS-related DEGs, revealing the down-regulated expression of most QS genes. (C) The schematic diagram of *P. aeruginosa* QS network and its downstream virulent factor-related genes, showing the 3 main QS systems: *las*, *pqs*, and *rhl*. Genes with decreased expression in the QS network are marked in blue. (D) Comparison of the QS regulatory gene expression between peptide-treated group and untreated group (*n* = 3). (E) Virulence-associated genes regulated by the QS network also showed significantly down-regulated gene expression (*n* = 3). TCA, tricarboxylic acid.

The reduction of PYO (generally as a marker to assess the QS behavior) secretion in previous experiments showed the possible effect of 20:80-Bu:DM against QS. Thus, we collected the mature biofilms exposed to 20:80-Bu:DM (lower than MBEC) and sent for RNA sequencing (RNA-seq) to analyze the existed impact on the QS pathway at the transcriptional level. The number of differentially expressed genes (DEGs) was counted and shown in the volcano plot (Fig. S9). There were a total of 3,320 genes significantly changed under 20:80-Bu:DM treatment; 726 were up-regulated (log_2_ fold change > 1), and 772 were down-regulated (log_2_ fold change < −1). The DEGs were all matched with the Kyoto Encyclopedia of Genes and Genomes (KEGG) pathway database for KEGG pathway mapping. The top 10 pathways with the most DEGs were screened out (Fig. S11A), containing biofilm formation and QS pathways that were vital to the environmental adaptability, virulence, and drug resistance of bacteria. According to the KEGG functional annotations, the DEGs participated in the biological pathway were classified into 5 main branches and several subcategories to draw the KEGG pathway classification map (Fig. S11B), and the distribution of QS-related genes was highlighted. We found that the QS-related DEGs most focused on the functional term of cellular processes–cellular community, revealing the occurred changes in bacterial communities caused by 20:80-Bu:DM. The KEGG pathway enrichment analysis (Fig. 5A) was further conducted to confirm, and, as expected, DEGs were significantly enriched in the above 2 pathways, indicating that 20:80-Bu:DM could affect the QS system, thereby interfering with the biofilm formation of the *P. aeruginosa*. The heatmap exhibits the down-regulated expression of the key QS genes (Fig. 5B). We studied 3 important QS systems including *las*, *rhl*, and *pqs*, and the considerably down-regulated gene expression was detected in different levels of the hierarchical QS network (Fig. 5C and D). The results were corresponding with the reduced production of the PYO, whose biosynthetic genes were coactivated by the *rhl* and *pqs* system [54,55], and the 2 QS systems were centrally regulated by the *las* system [56,57]. Meanwhile, the expression of various virulence-associated genes regulated by the QS network upstream such as *lasA*, *lasB*, *lecA*, *PA2569*, *phzABCDEFG*, *phnAB*, and *rhlAB* was also substantially down-regulated (Fig. 5C and E), demonstrating that the 20:80-Bu:DM treatment could reduce the virulence of the *P. aeruginosa*.

### 20:80-Bu:DM inhibits biofilms and cures *P. aeruginosa* pneumonia

**Fig. 6.**
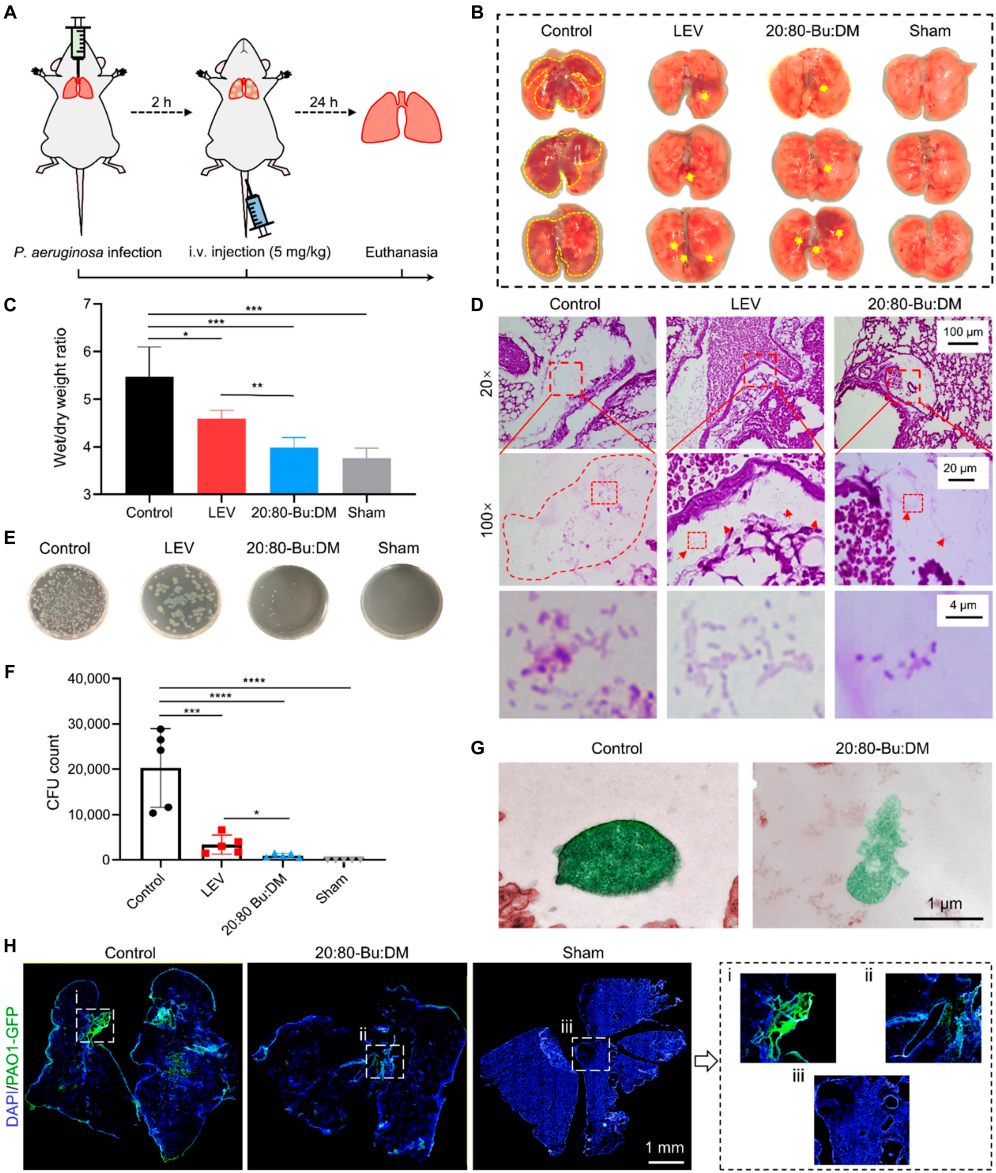
20:80-Bu:DM eradicates bacteria in vivo and inhibits biofilm colonization in *P. aeruginosa* pneumonia. (A) Illustration of the establishment of the bacterial pneumonia model and the administration process. (B) Photographs of the lungs taken out from the infection pneumonia mice with different drug treatments, showing the differences in morphology and pathological symptoms. (C) The wet/dry ratio of the lungs for tissue edema analysis and the 20:80-Bu:DM-treated group showed significantly reduced edema compared with the control and LEV-treated group (*n* = 3). (D) Viable bacteria in lungs were examined by plate counting method, and (E) the quantitative result showed that the 20:80-Bu:DM has a statistically significant increase in bacteria elimination efficiency in vivo compared with LEV (*n* = 3). (f) Gram-stained sections of lungs from infected mice. The bacterial cluster is marked with red boundaries and red arrows, and the 20:80-Bu:DM-treated lung shows reduced bacteria infiltration and accumulation in tissue. (G) TEM images of infiltrating bacteria in the lung. 20:80-Bu:DM-treated bacteria show morphological changes and content leakage corresponding with the in vitro characterization. (H) Lung cross-sections of PAO1-GFP strain-infected mice. The blue part is tissue stained with DAPI, and the green indicates the bacteria distribution. In the control group, a large number of bacteria attached to the bronchial wall will develop into a biofilm, and the 20:80-Bu:DM shows an inhibitory effect against biofilm formation in vivo. Significance of differences was determined using the one-way ANOVA method. *P < 0.05, **P < 0.01, ***P < 0.001, and ****P <0.0001. i.v., intravenous.

We investigated the therapeutic effect of 20:80-Bu:DM on the *P. aeruginosa* pneumonia model, and the process of model establishment and drug administration is shown in Fig. 6A. A sham surgery group was set to exclude the influence of the surgery, and a group treated with an equal dose of levofloxacin (LEV), a clinically used broad-spectrum antibiotic, was set for comparison. The lungs of mice were excised and photographed for observation (Fig. 6B). For the control group, swelling and extensive dark red areas (marked by yellow lines) appeared on the lungs, which meant hemorrhage and congestion. The pathologic symptoms were considerably alleviated for the LEV and 20:80-Bu:DM groups, in which the appearance of lungs was closer to the sham surgery group and showed a normal pink color. The pulmonary edema was determined by the wet/dry weight ratio of the excised lungs (Fig. 6C). The LEV and 20:80-Bu:DM treatment groups had significantly reduced edema compared with the control, and 20:80-Bu:DM seems to show a better healing effect than the LEV. We then enumerated the viable pathogens in the lungs to further evaluate the antimicrobial efficiency of 20:80-Bu:DM in vivo (Fig. 6D and E). Mice with LEV and 20:80-Bu:DM treatments showed a significant decrease in bacteria count in the lung. Moreover, the bacterial level in the 20:80-Bu:DM group was significantly lower compared with the LEV, indicating a higher efficiency of 20:80-Bu:DM against bacteria in *P. aeruginosa* pneumonia. The excised lungs were then sliced and stained with Gram staining to observe the bacteria inside (Fig. 6F and Fig. S12). For the control group, a large number of bacteria infiltrated and accumulated in the tissue, while being small clusters of bacteria in the LEV group and only a few dispersed bacteria in the 20:80-Bu:DM group. The bacterial morphology in vivo was analyzed by TEM images (Fig. 6G and Fig. S13). We found that bacteria in the lung of the 20:80-Bu:DM group had destroyed morphology and content leakage, which was substantially consistent with the appraisal in vitro.

With the bacterial penetration and proliferation in the lung, acute pneumonia might develop into a chronic one, and the biofilm would gradually form and colonize in the lung during the period [58–60]. To visualize the development of the lung infection, we used green fluorescent protein (GFP)-labeled *P. aeruginosa O1* (PAO1) to infect the mice for fluorescence imaging of the cross-sections through the lung (Fig. 6H). The bacteria distribution was indicated by the green fluorescence, and the tissue was stained with 4′,6-diamidino-2-phenylindole (DAPI) in blue. For the mice of the control group, the PAO1-GFP showed a widespread distribution in the whole lung, especially a massive attachment in the bronchial wall, which meant that the bacterial community was forming a biofilm lifestyle. The bacteria accumulation significantly decreased with 20:80-Bu:DM treatment, indicating the inhibitory effect of the 20:80-Bu:DM on biofilm formation in vivo.

### 20:80-Bu:DM alleviates the inflammatory response

**Fig. 7.**
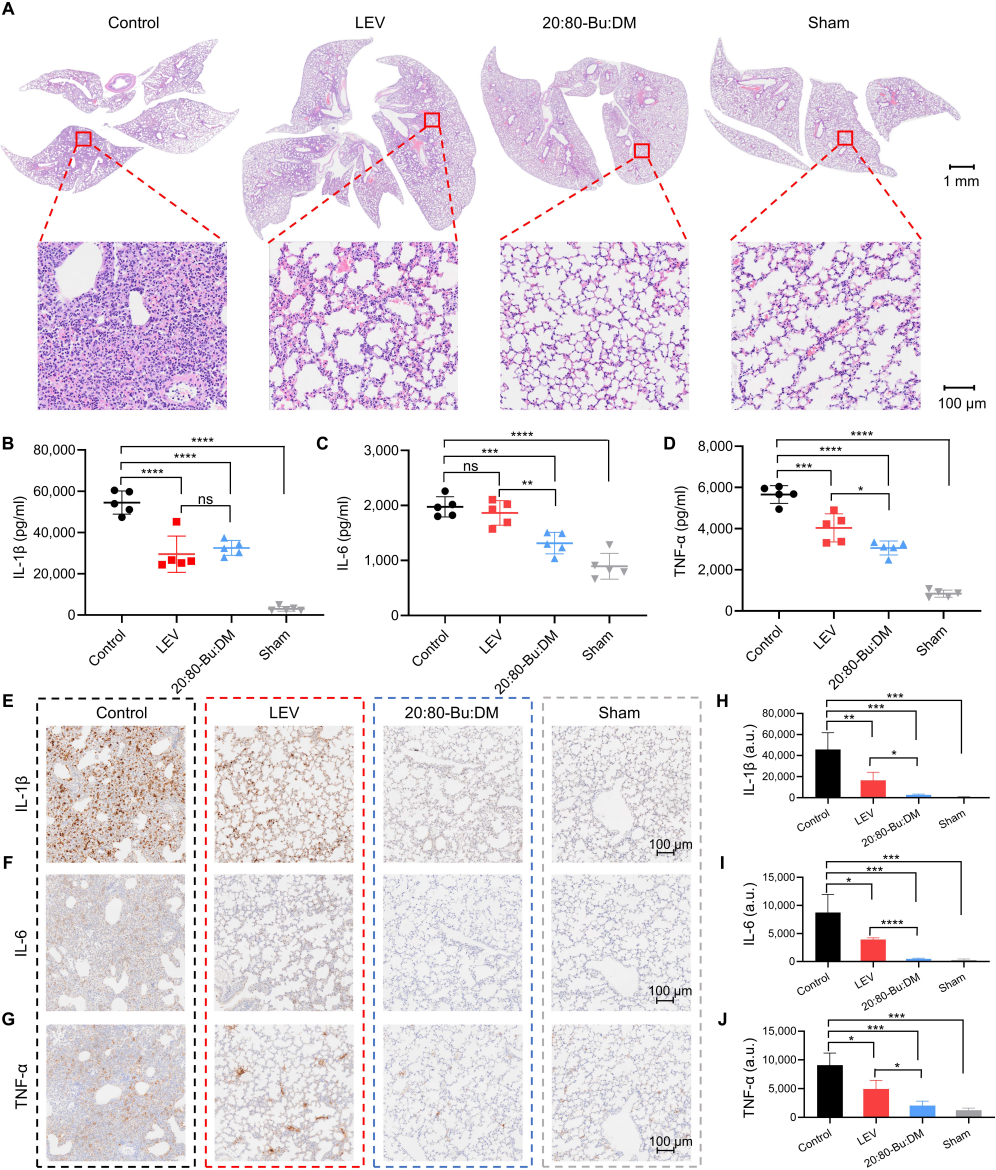
20:80-Bu:DM reduces inflammatory response in *P. aeruginosa* pneumonia. (A) H&E staining lung sections of infected mice. The 20:80-Bu:DM-treated mice show reduced inflammatory cells infiltration and histological injury (alveolar septal thickening, Intra-alveolar hemorrhage, hyaline membrane formation, etc.) compared with the LEV and control groups. (B to D) Inflammatory cytokine expression measured by ELISA, IL-1β, IL-6, and TNF-α shows decreased expression in the LEV- and 20:80-Bu:DM-treated groups, and IL-6 and TNF-α show a statistically significant reduction in the peptide-treated group compared with the LEV-treated group (*n* = 5). (E to G) Immunohistochemical analysis of lung sections. (H to J) Quantitative results of the immunohistochemical analysis, for IL-1β, IL-6, and TNF-α. The 20:80-Bu:DM-treated group shows a significant reduction compared with the LEV (*n* = 3). Significance of differences was determined using the one-way ANOVA method. *P < 0.05, **P < 0.01, ***P < 0.001, and ****P <0.0001.

The hematoxylin and eosin (H&E) staining was performed, and the overview and the typical images reflected varying degrees of inflammation in groups with different treatments (Fig. 7A). For mice without drug administration, extensive inflammatory cell infiltration and histological injury (alveolar septal thickening, intra-alveolar hemorrhage, hyaline membrane formation, etc.) were found in the lung. In contrast, LEV- and 20:80-Bu:DM-treated mice showed less inflammatory cell infiltration and reduced lung injury, and 20:80-Bu:DM even demonstrated a better inflammation alleviation effect compared with LEV. The homogenate of the lung was prepared for quantitative enzyme-linked immunosorbent assay (ELISA) of the inflammatory cytokines. We measured representative factors including interleukin-1β (IL-1β), IL-6, tumor necrosis factor-α (TNF-α), and IL-10 (Fig. 7B to D and Fig. S14A), which were all significantly decreased in the LEV- and 20:80-Bu:DM-treated groups. Notably, partial inflammatory factors (IL-6 and TNF-α) were found significantly lower in the 20:80-Bu:DM therapy than LEV treatment, indicating the clinical application potential of 20:80-Bu:DM. The inflammatory factors were further detected by immunohistological analysis of the lung sections. The overview images profiled the distribution of inflammatory cytokines in the lung (Figs. S14B and C and S15), and the enlarged shots showed the expression level of the cytokines under different treatments (Fig. 7E to G). The results were quantified for comparison (Fig. 7H to J). For IL-1β, IL-6, and TNF-α, the expression level in the 20:80-Bu:DM-treated group showed a significant decrease compared with the control and LEV groups. We also assessed myeloperoxidase (MPO), a reliable indicator of neutrophils (Fig. S16A and B) and obtained the same conclusion that 20:80-Bu:DM could alleviate the inflammatory response of the body when fighting against invading bacteria.

### 20:80-Bu:DM shows effectiveness to ECPLA

**Fig. 8.**
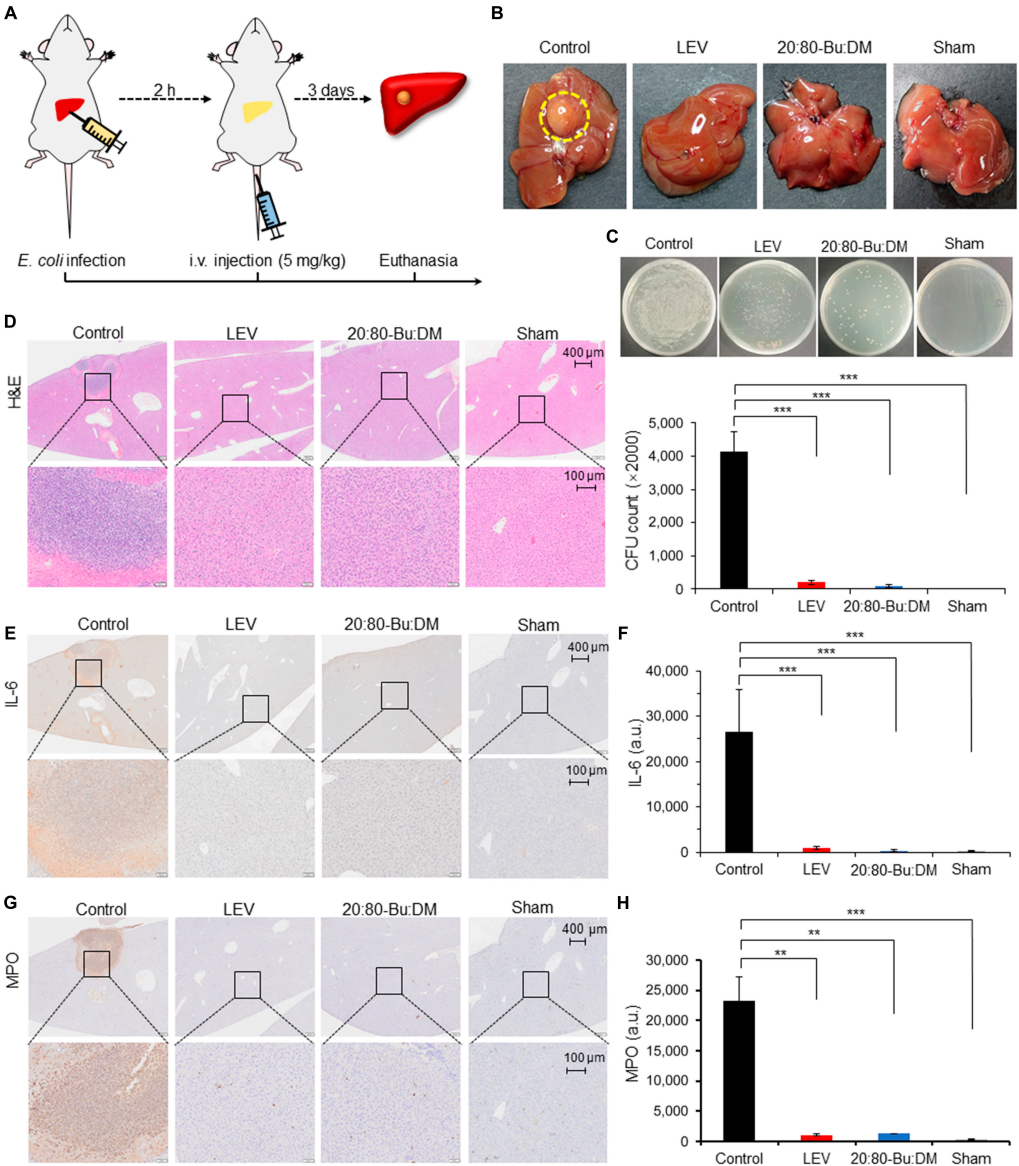
20:80-Bu:DM eradicates pyogenic liver abscess and reduces inflammatory response in ECPLA. (A) A illustration of the ECPLA model establishment and the administration process. (B) Photographs of the livers taken from ECPLA mice with different treatments. The liver abscess in the control is highlighted with a yellow circle. (C) Viable bacteria in livers of each group. The counted CFU number is shown in the bottom, and viable bacteria are significantly reduced for the 20:80-Bu:DM group (*n* = 3). (D) H&E-stained liver cross-sections show a large amount of inflammatory cell infiltration in the abscess, and there is no obvious inflammatory cells’ infiltration in the 20:80-Bu:DM group. (E and F) IL-6 expression level and distribution in livers of ECPLA mice. The quantitative results show a statistically significant difference between the control and the 20:80-Bu:DM (*n* = 3). (G and H) MPO expression level indicates the increase in neutrophils in the abscess site, and for the 20:80-Bu:DM group, the MPO expression showed no increase (*n* = 3). Significance of differences was determined using the one-way ANOVA method. **P < 0.01 and ***P < 0.001.

Pyogenic liver abscess is also a severe threat to human health and contributes to predominant morbidity and mortality historically. We further used ECPLA murine model to verify the effectiveness of 20:80-Bu:DM in *E. coli*-induced infection diseases, and the model establishment and drug administration process was followed as illustrated in Fig. 8A. Abscesses appeared in the livers of infected mice injected with phosphate-buffered saline (PBS), but no obvious purulent appearance emerged in livers of mice treated with 20:80-Bu:DM (Fig. 8B). The bacteria enumeration was performed and demonstrated outstanding antibacterial activities of the 20:80-Bu:DM, which was equivalent to LEV (Fig. 8C). The pathological changes and inflammation of livers were analyzed afterward using H&E staining (Fig. 8D). For the control, the abscesses area could be clearly distinguished in the liver section, and considerable inflammatory cell accumulation was also observed. On the contrary, no obvious inflammation was found in the liver sections of LEV and 20:80-Bu:DM groups. For chemokine response investigation, the control group showed significantly enhanced secretion of IL-6, MPO, and IL-1β; however, the 20:80-Bu:DM group had no significantly increased expression in these factors, indicating the excellent therapeutic effect of 20:80-Bu:DM for ECPLA (Fig. 8E to H and Fig. S17).

### In vivo biosafety evaluation of the 20:80-Bu:DM

**Fig. 9.**
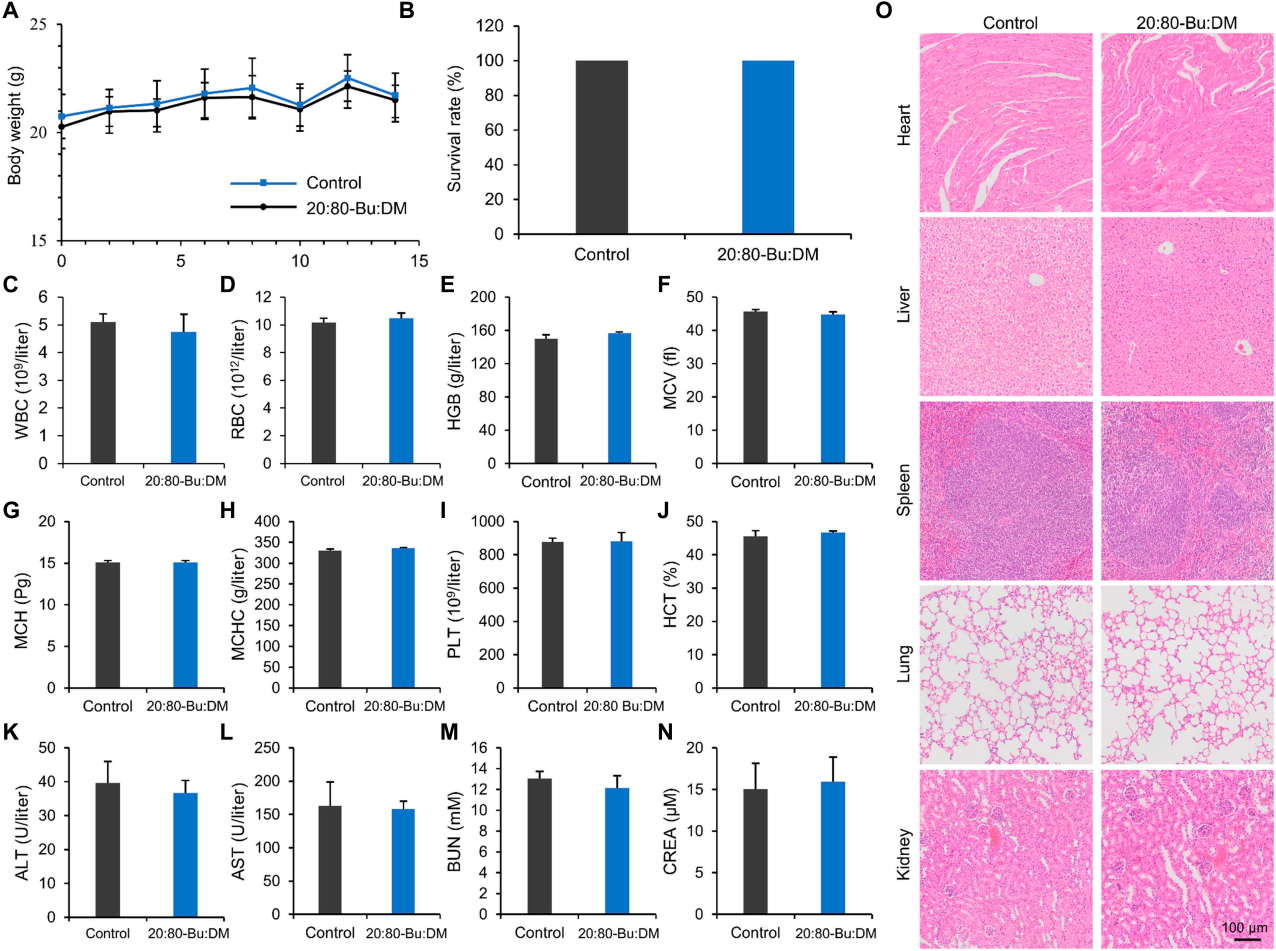
In vivo biosafety of the 20:80-Bu:DM. (A) Body-weight curve in 2 weeks of the mice intravenously injected with 20:80-Bu:DM (5 mg/kg) (*n* = 10). (B) Survival rate of the drug administered mice at day 14. (C to J) Hematology surveys of the mice show all parameters in a normal range, and there are no significant differences between the mice injected with PBS and 20:80-Bu:DM (*n* = 5). (K to N) The blood biochemical test shows the normal function of livers [alanine aminotransferase (ALT) and aspartate aminotransferase (AST)] and kidneys [blood urea nitrogen (BUN) and creatinine (CREA)] (*n* = 5). (O) H&E staining of the main organs, suggesting that there is no significant organ toxicity in the 20:80-Bu:DM-treated group. Significance of differences was determined using the Student’s *t* test.

A 14-day biosafety assessment for 20:80-Bu:DM was carried out in vivo. We found that the mice with intravenous injection of 20:80-Bu:DM (5 mg/kg) had no significant difference in body weight with the mice injected with PBS by a consecutive record in 14 days (Fig. 9A). Both groups of mice counted showed 100% survival 14 days later (Fig. 9B). For hematology surveys (Fig. 9C to J), the hematology parameters were all within the normal range and showed no significant difference between the groups, reflecting that the 20:80-Bu:DM has no obvious hematotoxicity at the administered dose. For blood biochemistry analysis, the liver function indexes aspartate aminotransferase and alanine aminotransferase and the kidney function indexes blood urea nitrogen and creatinine were at the normal level, proving that 20:80-Bu:DM has no obvious toxicity to the liver and kidney. For pathological analysis, the main organ slices stained with H&E showed no obvious lesions and inflammation in the sections of the 20:80-Bu:DM, indicating that no organ toxicity of the peptides appeared in 14 days (Fig. 9O). In summary, the 20:80-Bu:DM is validated biosafety in vivo, and it is promising for the drug to apply in clinical antimicrobial therapy.

## Conclusion

In this work, we developed the HDP-mimicking antimicrobial β-peptide polymer 20:80-Bu:DM with enhanced efficiency and stability against bacteria. The 20:80-Bu:DM could eradicate bacteria in bactericidal and QS interference dual modes, which is more effective to target bacteria of different lifestyles, including planktonic bacteria and biofilms. For planktonic bacteria, 20:80-Bu:DM has membrane-targeted antimicrobial activities that can destroy the bacterial membranes in an effective and fast way of conducting the cell lysis and content leakage and final death. For bacterial communities, 20:80-Bu:DM can penetrate deeply into biofilms and inhibit their QS system to block bacterial communication leading to bacterial virulence reduction. With QS interference and bactericidal dual modes, 20:80-Bu:DM can effectively eradicate mature biofilms. For *P. aeruginosa* pneumonia and ECPLA in vivo, the 20:80-Bu:DM is effective to eradicate invasive bacteria in tissue and, in the meantime, alleviate the excessive inflammation of the body. Moreover, 20:80-Bu:DM can inhibit biofilm formation in vivo, preventing persistent bacterial infection. Together, 20:80-Bu:DM shows great potential as a promising antimicrobial agent for clinical translation, owing to its’ potent therapeutic efficacy and negligible toxicity both in vitro and in vivo.

## Methods

### Bacterial strains and culture growth assay

Bacteria strains *E. coli* (ATCC 25922) were purchased from American Type Culture Collection. Strains of *P. aeruginosa* PAO1 and *P. aeruginosa* PAO1-GFP were acquired from the Second Affiliated Hospital of Zhejiang University. The frozen cells were activated in Luria–Bertani (LB) agar plate at 37 °C and then overnight grown in fresh LB liquid medium for proliferation. All experimental steps were performed under sterile conditions.

### Antibacterial analysis in vitro

Bacteria grown in LB for 9 h were diluted in Mueller–Hinton (MH) medium to 2 × 10^5^ CFU/ml. 20:80-Bu:DM and magainin II were diluted to concentrations ranging from 3.13 to 400 μg/ml via a 2-fold gradient dilution using MH medium and mixed with bacterial suspension with a volume ratio of 1:1. The mixture was added into a 96-well plate (100 μl per well) and incubated at 37 °C for 9 h, and the optical density value of each well was obtained at 600 nm on a SpectraMax M2 plate reader (*n* = 3). Wells containing MH medium only and wells containing cells in MH medium without polymer were included in the same plate as blank and positive control, respectively. The percentage of bacterial cells survival was calculated as follow:

$$\left(\%\text{cell growth}=\frac{A_{600}^{\text{polymer}}–A_{600}^{\text{blank}}}{A_{600}^{\text{control}}–A_{600}^{\text{blank}}}\times 100\right)$$

The MIC was defined as the minimum concentration of antibacterial agents to completely inhibit bacterial growth.

For simulating physiological conditions, MH medium containing 10% FBS was used for the antibacterial test. For antiproteolysis testing, 20:80-Bu:DM and magainin II were mixed with trypsin respectively at a ratio of 1:100 (w/w) and incubated at 37 °C for 40 min. Before MIC testing, the samples were heated for 10 min at 90 °C for protease inactivation (*n* = 3).

For the antibacterial test with higher bacterial concentrations, the bacteria were diluted in MH medium to a cell density at 2 × 10^7^ or 2 × 10^8^ CFU/ml (*n* = 3).

### SEM and TEM images and live/dead staining for the evaluation

For SEM characterization, PAO1 working suspension at a concentration of 10^7^ CFU/ml was treated with 20:80-Bu:DM (12.5 μg/ml) for 30 min. Bacteria were collected from centrifugation, washed with PBS, and then fixed using 4% glutaraldehyde in PBS at room temperature overnight. The fixed bacteria were washed with PBS once and sequentially dehydrated with gradient ethanol (30%, 50%, 70%, 80%, 90%, 95%, and 100%). Then, the sample was dried in air and used directly for field-emission scanning electron microscopy characterization (Hitachi S-4800). For TEM characterization, PAO1 (10^8^ CFU/ml) was incubated with 20:80-Bu:DM (100 μg/ml) at 37 °C. After 6 h, the bacteria were harvested and washed twice with PBS by centrifugation at 8,000 rpm for 5 min. PAO1 was fixed with 2.5% glutaraldehyde in PBS at 4 °C for 24 h and sent to the Analysis Center of Agrobiology and Environmental Sciences and Institute of Agrobiology and Environmental Sciences, Zhejiang University (Zhejiang, China) for observation (Hitachi H-7650, Japan). SYTO 9/PI double-stained kit (L7012 LIVE/DEAD BacLight Bacterial Viability Kit, Thermo Fisher Scientific, USA) was used to stain the treated bacteria. Fluorescent images were photographed by the confocal laser scanning microscope (Leica TCS SP8, Germany).

### Time-lapse fluorescent confocal imaging of bacteria

Mid-logarithmic phase PAO1 was diluted in MH medium to 2 × 10^7^ CFU/ml. Ten microliters of PAO1 working suspension in MH was dropped onto a glass-bottomed culture dish and was kept still for 10 min. Dye-20:80-Bu:DM (50 μg/ml; in MH medium) was mixed with PI (80 μg/ml; in MH medium) at 1:1 (v/v) ratio, and then 10 μl of the solution was added into the bacteria drop, followed by immediate microscope imaging of bacteria on a 30-s time scale using 3 channels, the bright field of differential interference contrast, 488 nm of detecting dye-20:80-Bu:DM, and 562 nm of detecting PI.

### Visualization of the bacterial membrane disruption

PAO1 grown to logarithmic phase was mixed with FM 4-64 (10 μg/ml) at a ratio of 1:1. For 10 min of incubation on ice, the suspension was dropped into the glass bottom of a confocal petri dish and settled statically for 10 min. Dye-20:80-Bu:DM (50 μg/ml) was added at the ratio of 1:1, and the photographs were immediately taken (Leica TCS SP8, Germany). The rest of the suspension was incubated with dye-20:80-Bu:DM (25 μg/ml) at 4 °C for 12 h. Bacteria stained with FM 4-64 but without dye-20:80-Bu:DM were served as control.

### Evaluation of antibiofilm effects of the 20:80-Bu:DM

For the biofilm fluorescence imaging analysis, the planktonic bacteria were grown in the 6-well plate [PAO1 (10^5^ CFU/ml) was seeded in 2 ml of LB, and for confocal analysis, the biofilm was formed in a piece of coverslip] for 2 days to allow the biofilm formation. The mature biofilm was treated with PBS or 20:80-Bu:DM (0, 25, 50, 100, and 200 μg/ml) for 24 h, respectively. The supernatant was separated for PYO extraction. The biofilm was washed with PBS and stained with SYTO 9 for observation. The bacteria density and thickness of the biofilm were quantified with the confocal laser scanning microscope.

The PYO quantification assay was conducted by extraction with chloroform and hydrochloric acid (HCl) orderly. A total of 50% total volume of chloroform was added to the culture. The sample was vortexed vigorously for 30 s and then settled for 10 min. We discarded the upper layer (aqueous phase) and carefully transferred the chloroform layer (blueish portion) into a fresh tube. Twenty percent of the total volume of 0.2 M HCl was added and thoroughly mixed with the extraction by vortex oscillation. After 15 min of the settlement, the aqueous phase turned to pink and the blue chloroform fraction became clear. The upper layer (aqueous fraction) was the extracted PYO and its absorbance at 520 nm was measured for quantification.

For MBEC evaluation, biofilms were formed in a 96-well plate and treated with different concentrations of 20:80-Bu:DM (0, 25, 50, 100, 200, and 400 μg/ml) for 24 h. Subsequently, the biofilms were washed twice and collected in 1 ml of PBS for ultrasonication. The bacterial number was counted by spread plate.

### Animals

Pneumonia and pyogenic liver abscess models were established with 7-week BALB/c mice purchased from Shanghai SLAC Laboratory Animal Co. Ltd. (Shanghai, China). The whole animal procedures in this study were obtained approval of the Institutional Animal Care and Use Committee of Zhejiang University.

### Bacterial pneumonia models

For the pathogen preparation, PAO1 grown to the logarithmic phase was centrifuged and washed twice with PBS. The harvested bacteria were resuspended with PBS to the concentration of 5 × 10^6^ CFU/ml, and the bacterial suspension was put on ice to keep active. The animals were anesthetized by airway inhalation with isoflurane and then tracheotomized. Subsequently, 50 μl of the PAO1 was intratracheally injected into the lung using the insulin needle. For the mice of the sham surgery group, 50 μl of PBS was injected intratracheally instead of the bacterial suspension. Then, the incision was sutured with surgical staples, and anesthesia was removed. The postoperative mice were kept warm and woke up about 10 min later. Two hours after the PAO1 challenge, 200 μl of 20:80-Bu:DM (500 μg/ml) or LEV (500 μg/ml) was injected intravenously into the mice, respectively. Twenty-four hours later, the mice were euthanized, and the whole lung of each mouse was excised for analysis of morphology, histology, pathology, quantitative bacteriology, and immunology. The morphological observation was performed with a stereomicroscope (Leica S9i, German).

### Pulmonary edema measurement

Pulmonary edema of the infected lungs was evaluated with the lung wet-to-dry weight ratio. The freshly excised lungs were immediately weighed, and the values were recorded as the wet weight. Subsequently, the lungs were placed into an oven at 60 °C for 48 h. Weights of the dried lungs were measured, and the values were served as the dry weight.

### Bacteriological evaluation

The infected lung was obtained 24 h after treatment for bacteriological evaluation. For bacterial enumeration, the freshly excised whole lung was added into 1.5 ml of a sterile tube (Eppendorf, Germany), and 500 μl of the PBS was added to homogenize (TissuePrep, China). One hundred microliters of the tissue homogenates were diluted to 1,000 μl of PBS, and 100 μl of the mixture was taken for the plate-counting method (*n* = 5). For TEM imaging, the excised lungs were fixed with 2.5% glutaraldehyde in PBS at 4 °C for 24 h. Then, the samples were cut into small pieces (1 mm × 3 mm × 1 mm) and sent for observation. For the Gram staining method, the lungs were fixed with formalin, embedded in paraffin, and finally sliced for the Gram staining.

### Fluorescent imaging of PAO1 lung infection

The PAO1-GFP strain was used to establish the lung infection model. Twenty-four hours later, the lungs were excised and embedded in optimal cutting temperature compound and frozen at −80 °C. The frozen tissue was sliced in a cryostat (Leica CM1950, Germany). The frozen lung sections were stained by DAPI and photographed by the Operetta CLS High-Content Analysis System (PerkinElmer, USA).

### Inflammation analysis

The cytokine levels in lung homogenates (24 h after infection) were measured by ELISA (Boster Biological Technology Co. Ltd., Wuhan, China). The lung homogenates were prepared as per the above protocol and centrifuged at 4 °C (10,000*g* for 10 min). The supernatants were separated out and saved in −80 °C. When performing the ELISA, samples were previously thawed on ice for 20 min. Meanwhile, the lung sections (24 h after infection) were prepared for H&E staining and immunohistochemical analysis.

### Pyogenic liver abscess models

The infectious *E. coli* suspension was prepared as follows: Logarithmic phase *E. coli* were harvested and washed twice by centrifugation, resuspended with PBS, and diluted to the concentration of 2 × 10^8^ CFU/ml. The pyogenic liver abscess model was established following the previous report. A transverse incision was made in the abdomen of the anesthetic mouse to expose the liver. Then, 50 μl of the bacterial suspension was injected into the liver parenchyma, and the incision was sutured. One hour later, the mice were randomly divided into 3 groups and treated with 200 μl of PBS, LEV (500 μg/ml), and 20:80-Bu:DM (500 μg/ml), respectively. The mice were sacrificed after 3 days, and the livers were harvested. For bacterial enumeration, livers were homogenized and diluted with PBS. The plate-counting method was used to evaluate the bacterial number. The livers were fixed and cut into tissue sections. H&E staining, Gram staining, and immunohistochemical staining were performed for histological and pathological analysis.

### In vivo biosafety evaluation

Healthy mice were injected intravenously with 200 μl of 20:80-Bu:DM (500 μg/ml). Body weight of the mice was consecutively measured for 2 weeks, and the survival rate was calculated. The blood of mice was collected, the plasma was sent for a hematology survey (*n* = 5), and the serum was separated out for biochemistry analysis (*n* = 5). After the mice were euthanized, the main organs were taken from the mice and processed to sections for H&E staining.

### Statistics

The data of RNA-seq were analyzed by OriginLab 2020, and the other statistics in this study were all analyzed by GraphPad Prism 8. The data in experiments were all presented as means ± SD with error bars. The statistical differences for multiple comparisons were analyzed by the one-way analysis of variance (ANOVA) method, and all the data were tested to be homogeneous before the one-way ANOVA analysis. For the in vivo biosafety evaluation, the significance of differences between the 2 groups was determined by the double-tailed Student's *t* test. The significant differences were shown as the following: *P < 0.05, **P < 0.01, ***P < 0.001, and ****P < 0.0001.

For RNA-seq, the data were analyzed by the double-tailed Student's *t* test, followed by multiple test correction by the Benjamini–Hochberg method. The data were input into the KEGG database to calculate the gene number in different pathways, and the analysis tool, KEGG mapper, was used to draw the classification map. The KOBAS software (3.0) was used to statistically analyze the enrichment of DEGs in KEGG pathways.

## Data Availability

All data needed in the paper are present in the paper and in the Supplementary Materials. Additional data related to this paper may be requested from the authors.

## Supplementary Materials

Supplementary 1 Fig. S1. Antimicrobial activities and stability of the 20:80-Bu:DM. Fig. S2. Cytotoxicity of the 20:80-Bu:DM in vitro. Fig. S3. PAO1 treated with 20:80-Bu:DM stained with Syto 9/PI double staining kit, showing 93% cell death compared with the untreated group. Fig. S4. 1H NMR spectrum of dye initiator in CDCl_3_, 600 MHz. Fig. S5. 13C NMR spectrum of dye initiator in CDCl_3_, 151 MHz. Fig. S6. HREI-MS spectrum of dye initiator. Fig. S7. 1H NMR spectrum of dye-poly-β-peptide, dye-20:80-Bu:DM. Fig. S8. Visualize the process of bacterial membrane damage. Fig. S9. TEM images of the PAO1 treated with PBS and 20:80-Bu:DM respectively. Fig. S10. Volcano plot of gene expression differences between *P. aeruginosa* biofilms and biofilms treated with 20:80-Bu:DM. Fig. S11. DEG distribution in QS-related pathway. Fig. S12. Gram staining of the lung sections, indicating the tissue distribution and the bacterial cluster size in the lungs of mice with different treatments. Fig. S13. TEM images of the PAO1 in the lung, showing that *P. aeruginosa* in vivo is disrupted by 20:80-Bu:DM. Fig. S14. IL-10 expression level in the lung of mice with PAO1 infection. Fig. S15. Overview images showed expression level and distribution of the secreted inflammatory factors (IL-1β, IL-6, and TNF-α) in the lungs of mice with different treatments, and the inflammation in the 20:80-Bu:DM group showed significant reduction. Fig. S16. Fig. S17. Fig. S18. ^1^H NMR spectrum of compound 1 in CDCl_3_, 400 MHz. Fig. S19. ^13^C NMR spectrum of compound 1 in CDCl_3_, 100 MHz. Fig. S20. HREI-MS spectrum of compound 1.

## References

[1] Kallander K, Burgess DH, Qazi SA. Early identification and treatment of pneumonia: A call to action. Lancet Glob Health. 2016;4(1):e12–e13. 26577842 10.1016/S2214-109X(15)00272-7 PMC5357734

[2] Shann F. Bacterial pneumonia: Commoner than perceived. Lancet. 2001;357(9274):2070–2072. 10.1016/S0140-6736(00)05226-0 11445094

[3] Gu D, Dong N, Zheng Z, Lin D, Huang M, Wang L, Chan EWC, Shu L, Yu J, Zhang R, et al. A fatal outbreak of ST11 carbapenem-resistant hypervirulent Klebsiella pneumoniae in a Chinese hospital: A molecular epidemiological study. Lancet Infect Dis. 2018;18:37–46. 28864030 10.1016/S1473-3099(17)30489-9

[4] Swanson MS. Bacterial pathogenesis: An angel of death fights infection. Nat Microbiol. 2016;1:16012. 27572172 10.1038/nmicrobiol.2016.12

[5] Brown ED, Wright GD. Antibacterial drug discovery in the resistance era. Nature. 2016;529:336–343. 26791724 10.1038/nature17042

[6] Di YP, Lin Q, Chen C, Montelaro RC, Doi Y, Deslouches B. Enhanced therapeutic index of an antimicrobial peptide in mice by increasing safety and activity against multidrug-resistant bacteria. Sci Adv. 2020;6:eaay6817. 32426473 10.1126/sciadv.aay6817 PMC7195177

[7] Bloom DE, Black S, Salisbury D, Rappuoli R. Antimicrobial resistance and the role of vaccines. Proc Natl Acad Sci USA. 2018;115:12868–12871. 30559204 10.1073/pnas.1717157115 PMC6305009

[8] Villa F, Villa S, Gelain A, Cappitelli F. Sub-lethal activity of small molecules from natural sources and their synthetic derivatives against biofilm forming nosocomial pathogens. Curr Top Med Chem. 2013;13:15680266. 10.2174/15680266113136660225 24200356

[9] Klümper U, Recker M, Zhang L, Yin X, Zhang T, Buckling A, Gaze WH. Selection for antimicrobial resistance is reduced when embedded in a natural microbial community. ISME J. 2019;13:2927–2937. 31384011 10.1038/s41396-019-0483-z PMC6864104

[10] Yurtsev EA, Chao HX, Datta MS, Artemova T, Gore J. Bacterial cheating drives the population dynamics of cooperative antibiotic resistance plasmids. Mol Syst Biol. 2013;9:683. 23917989 10.1038/msb.2013.39 PMC3779801

[11] Jørgensen PS, Aktipis A, Brown Z, Carrière Y, Downes S, Dunn RR, Epstein G, Frisvold GB, Hawthorne D, Gröhn YT, et al. Antibiotic and pesticide susceptibility and the Anthropocene operating space. Nat Sustain. 2018;1:632–641.

[12] Dandekar AA, Chugani S, Greenberg EP. Bacterial quorum sensing and metabolic incentives to cooperate. Science. 2012;338:264–266. 23066081 10.1126/science.1227289 PMC3587168

[13] Curran CS, Bolig T, Torabi-Parizi P. Mechanisms and targeted therapies for *pseudomonas aeruginosa* lung infection. Am J Respir Crit Care Med. 2018;197:708–727. 29087211 10.1164/rccm.201705-1043SO PMC5855068

[14] Yang C, Luo Y, Lin H, Ge M, Shi J, Zhang X. Niobium carbide mXene augmented medical implant elicits bacterial infection elimination and tissue regeneration. ACS Nano. 2021;15:1086–1099. 33372766 10.1021/acsnano.0c08045

[15] Bjarnsholt T, Ciofu O, Molin S, Givskov M, Høiby N. Applying insights from biofilm biology to drug development-can a new approach be developed? Nat Rev Drug Discov. 2013;12:791–808. 24080700 10.1038/nrd4000

[16] Wu H, Moser C, Wang H-Z, Høiby N, Song Z-J. Strategies for combating bacterial biofilm infections. Int J Oral Sci. 2015;7(1):1–7. 25504208 10.1038/ijos.2014.65 PMC4817533

[17] Kim J, Kwon J, Kim M, Do J, Lee D, Han H. Low-dielectric-constant polyimide aerogel composite films with low water uptake. Polym J. 2016;48:829–834.

[18] Hennemann LC, Nguyen D. LasR-regulated proteases in acute vs. chronic lung infection: A double-edged sword. Microb Cell. 2021;8(7):161–163. 34250084 10.15698/mic2021.07.755 PMC8246023

[19] Ozer EA, Pezzulo A, Shih DM, Chun C, Furlong C, Lusis AJ, Greenberg EP, Zabner J. Human and murine paraoxonase 1 are host modulators of *Pseudomonas aeruginosa* quorum-sensing. FEMS Microbiol Lett. 2005;253:29–37. 16260097 10.1016/j.femsle.2005.09.023

[20] Pearson JP, Feldman M, Iglewski BH, Prince A. *Pseudomonas aeruginosa* cell-to-cell signaling is required for virulence in a model of acute pulmonary infection. Infect Immun. 2000;68:4331–4334. 10858254 10.1128/iai.68.7.4331-4334.2000 PMC101761

[21] Lesprit P, Faurisson F, Join-Lambert O, Roudot-Thoraval F, Foglino M, Vissuzaine C, Carbon C. Role of the quorum-sensing system in experimental pneumonia due to *Pseudomonas aeruginosa* in rats. Am J Respir Crit Care Med. 2003;167(11):1478–1482. 12569080 10.1164/rccm.200207-736BC

[22] Song T, Duperthuy M, Wai S. Sub-optimal treatment of bacterial biofilms. Antibiotics. 2016;5(2):23. 27338489 10.3390/antibiotics5020023 PMC4929437

[23] Liu X, Liu F, Ding S, Shen J, Zhu K. Sublethal levels of antibiotics promote bacterial persistence in epithelial cells. Adv Sci. 2020;7:1900840. 10.1002/advs.201900840 PMC7509632 32999821

[24] Andersson DI, Hughes D. Microbiological effects of sublethal levels of antibiotics. Nat Rev Microbiol. 2014;12:465–478. 24861036 10.1038/nrmicro3270

[25] Wistrand-Yuen E, Knopp M, Hjort K, Koskiniemi S, Berg OG, Andersson DI. Evolution of high-level resistance during low-level antibiotic exposure. Nat Commun. 2018;9:1599. 29686259 10.1038/s41467-018-04059-1 PMC5913237

[26] Koo H, Allan RN, Howlin RP, Stoodley P, Hall-Stoodley L. Targeting microbial biofilms: Current and prospective therapeutic strategies. Nat Rev Microbiol. 2017;15:740–755. 28944770 10.1038/nrmicro.2017.99 PMC5685531

[27] De Santis E, Alkassem H, Lamarre B, Faruqui N, Bella A, Noble JE, Micale N, Ray S, Burns JR, Yon AR, et al. Antimicrobial peptide capsids of de novo design. Nat Commun. 2017;8(1):2263. 29273729 10.1038/s41467-017-02475-3 PMC5741663

[28] Puthia M, Butrym M, Petrlova J, Strömdahl A-C, Andersson MÅ, Kjellström S, Schmidtchen A. A dual-action peptide-containing hydrogel targets wound infection and inflammation. Sci Transl Med. 2020;12:eaax6601. 31894104 10.1126/scitranslmed.aax6601

[29] Mookherjee N, Anderson MA, Haagsman HP, Davidson DJ. Antimicrobial host defence peptides: Functions and clinical potential. Nat Rev Drug Discov. 2020;19:311–332. 32107480 10.1038/s41573-019-0058-8

[30] Hancock REW, Alford MA, Haney EF. Antibiofilm activity of host defence peptides: Complexity provides opportunities. Nat Rev Microbiol. 2021;19:786–797. 34183822 10.1038/s41579-021-00585-w

[31] Lázár V, Martins A, Spohn R, Daruka L, Grézal G, Fekete G, Számel M, Jangir PK, Kintses B, Csörgő B, et al. Antibiotic-resistant bacteria show widespread collateral sensitivity to antimicrobial peptides. Nat Microbiol. 2018;3:718–731. 29795541 10.1038/s41564-018-0164-0 PMC6544545

[32] Bai S, Wang J, Yang K, Zhou C, Xu Y, Song J, Gu Y, Chen Z, Wang M, Shoen C, et al. A polymeric approach toward resistance-resistant antimicrobial agent with dual-selective mechanisms of action. Sci Adv. 2021;7:eabc9917. 33571116 10.1126/sciadv.abc9917 PMC7840121

[33] Wu Y, Zhang D, Ma P, Zhou R, Hua L, Liu R. Lithium hexamethyldisilazide initiated superfast ring opening polymerization of alpha-amino acid *N*-carboxyanhydrides. Nat Commun. 2018;9:5297. 30546065 10.1038/s41467-018-07711-y PMC6294000

[34] Zhou M, Qian Y, Xie J, Zhang W, Jiang W, Xiao X, Chen S, Dai C, Cong Z, Ji Z, et al. Poly(2-oxazoline)-based functional peptide mimics: Eradicating MRSA infections and persisters while alleviating antimicrobial resistance. Angew Chem. 2020;132:6354–6354. 10.1002/anie.202000505 32083767

[35] Zhou M, Jiang W, Xie J, Zhang W, Ji Z, Zou J, Cong Z, Xiao X, Gu J, Liu R. Peptide-mimicking poly(2-oxazoline)s displaying potent antimicrobial properties. Chem Med Chem. 2021;16:309–315. 32926562 10.1002/cmdc.202000530

[36] Chin W, Zhong G, Pu Q, Yang C, Lou W, De Sessions PF, Periaswamy B, Lee A, Liang ZC, Ding X, et al. A macromolecular approach to eradicate multidrug resistant bacterial infections while mitigating drug resistance onset. Nat Commun. 2018;9:917. 29500445 10.1038/s41467-018-03325-6 PMC5834525

[37] Xiong M, Lee MW, Mansbach RA, Song Z, Bao Y, Peek RM, Yao C, Chen LF, Ferguson AL, Wong GCL, et al. Helical antimicrobial polypeptides with radial amphiphilicity. Proc Natl Acad Sci USA. 2015;112:13155–13160. 26460016 10.1073/pnas.1507893112 PMC4629321

[38] Niu Y, Padhee S, Wu H, Bai G, Qiao Q, Hu Y, Harrington L, Burda WN, Shaw LN, Cao C, et al. Lipo-γ-AApeptides as a new class of potent and broad-spectrum antimicrobial agents. J Med Chem. 2012;55:4003–4009. 22475244 10.1021/jm300274p

[39] Zhang K, Du Y, Si Z, Liu Y, Turvey ME, Raju C, Keogh D, Ruan L, Jothy SL, Reghu S, et al. Enantiomeric glycosylated cationic block co-beta-peptides eradicate *Staphylococcus aureus* biofilms and antibiotic-tolerant persisters. Nat Commun. 2019;10:4792. 31636263 10.1038/s41467-019-12702-8 PMC6803644

[40] Degrado SJ, Mizutani H, Hoveyda AH. Modular peptide-based phosphine ligands in asymmetric catalysis: Efficient and enantioselective Cu-catalyzed conjugate additions to five-, six-, and seven-membered cyclic enones. J Am Chem Soc. 2001;123:755–756. 11456598 10.1021/ja003698p

[41] Mowery BP, Lindner AH, Weisblum B, Stahl SS, Gellman SH. Structure-activity relationships among random nylon-3 copolymers that mimic antibacterial host-defense peptides. J Am Chem Soc. 2009;131:9735–9745. 19601684 10.1021/ja901613g

[42] Chen S, Shao X, Xiao X, Dai Y, Wang Y, Xie J, Jiang W, Sun Y, Cong Z, Qiao Z, et al. Host defense peptide mimicking peptide polymer exerting fast, broad spectrum, and potent activities toward clinically isolated multidrug-resistant bacteria. ACS Infect Dis. 2020;6:479–488. 31922723 10.1021/acsinfecdis.9b00410

[43] Zhang Q, Ma P, Xie J, Zhang S, Xiao X, Qiao Z, Shao N, Zhou M, Zhang W, Dai C, et al. Host defense peptide mimicking poly-β-peptides with fast, potent and broad spectrum antibacterial activities. Biomater Sci. 2019;7:2144–2151. 30882803 10.1039/c9bm00248k

[44] Frackenpohl J, Arvidsson PI, Schreiber JV, Seebach D. The outstanding biological stability of β- and γ-peptides toward proteolytic enzymes: An in vitro investigation with fifteen peptidases. Chembiochem. 2001;2:445–455. 11828476 10.1002/1439-7633(20010601)2:6<445::aid-cbic445>3.3.co;2-i

[45] Sezen B, Franz R, Sames D. C−C Bond formation via C−H bond activation: Catalytic arylation and alkenylation of alkane segments. J Am Chem Soc. 2006;128:8364–8364. 10.1021/ja027891q 12418875

[46] Zhou R, Wu Y, Chen K, Zhang D, Chen Q, Zhang D, She Y, Zhang W, Liu L, Zhu Y, et al. A polymeric strategy empowering vascular cell selectivity and potential application superior to extracellular matrix peptides. Adv Mater. 2022;34:2200464–2200413. 10.1002/adma.202200464 36047924

[47] Melo MN, Ferre R, Castanho MARB. Antimicrobial peptides: Linking partition, activity and high membrane-bound concentrations. Nat Rev Microbiol. 2009;7:245–250. 19219054 10.1038/nrmicro2095

[48] Kalsy M, Tonk M, Hardt M, Dobrindt U, Zdybicka-Barabas A, Cytrynska M, Vilcinskas A, Mukherjee K. The insect antimicrobial peptide cecropin A disrupts uropathogenic *Escherichia coli* biofilms. NPJ Biofilms Microbiomes. 2020;6:6. 32051417 10.1038/s41522-020-0116-3 PMC7016129

[49] Lehrer RI, Barton A, Daher KA, Harwig SS, Ganz T, Selsted ME. Interaction of human defensins with *Escherichia coli*. Mechanism of bactericidal activity. J Clin Invest. 1989;84:553–561. 2668334 10.1172/JCI114198 PMC548915

[50] Borchers A, Pieler T. Programming pluripotent precursor cells derived from *Xenopus* embryos to generate specific tissues and organs. Genes. 2010;1:413–426. 24710095 10.3390/genes1030413 PMC3966229

[51] Benoit DSW, Sims KR, Fraser D. Nanoparticles for oral biofilm treatments. ACS Nano. 2019;13:4869–4875. 31033283 10.1021/acsnano.9b02816 PMC6707515

[52] Li X, Yeh YC, Giri K, Mout R, Landis RF, Prakash YS, Rotello VM. Control of nanoparticle penetration into biofilms through surface design. Chem Commun. 2015;51:282–285. 10.1039/c4cc07737g PMC4258172 25407407

[53] Gao Y, Wang J, Chai M, Li X, Deng Y, Jin Q, Ji J. Size and charge adaptive clustered nanoparticles targeting the biofilm microenvironment for chronic lung infection management. ACS Nano. 2020;14:5686–5699. 32320228 10.1021/acsnano.0c00269

[54] Chávez-Fuentes P, Ruiz-Marin A, Canedo-López Y. Biodiesel synthesis from *Chlorella vulgaris* under effect of nitrogen limitation, intensity and quality light: Estimation on the based fatty acids profiles. Mol Biol Rep. 2018;45:1145–1154. 30109546 10.1007/s11033-018-4266-9

[55] Yan R, Hu S, Ma N, Song P, Liang Q, Zhang H, Li Y, Shen L, Duan K, Chen L. Regulatory effect of DNA topoisomerase I on T3SS activity, antibiotic susceptibility and quorum- sensing-independent pyocyanin synthesis in *Pseudomonas aeruginosa*. Int J Mol Sci. 2019;20:1116. 30841529 10.3390/ijms20051116 PMC6429228

[56] Lee J, Wu J, Deng Y, Wang J, Wang C, Wang J, Chang C, Dong Y, Williams P, Zhang LH. A cell-cell communication signal integrates quorum sensing and stress response. Nat Chem Biol. 2013;9:339–343. 23542643 10.1038/nchembio.1225

[57] Lee J, Zhang L. The hierarchy quorum sensing network in *Pseudomonas aeruginosa*. Protein Cell. 2015;6:26–41. 25249263 10.1007/s13238-014-0100-x PMC4286720

[58] Sainz-Mejías M, Jurado-Martín I, McClean S. Understanding *Pseudomonas aeruginosa*–host interactions: The ongoing quest for an efficacious vaccine. Cell. 2020;9:2617. 10.3390/cells9122617 PMC7762141 33291484

[59] Broder UN, Jaeger T, Jenal U. LadS is a calcium-responsive kinase that induces acute-to-chronic virulence switch in *Pseudomonas aeruginosa*. Nat Microbiol. 2017;2:16184. 10.1038/nmicrobiol.2016.184 27775685

[60] Mauch RM, Jensen PØ, Moser C, Levy CE, Høiby N. Mechanisms of humoral immune response against *Pseudomonas aeruginosa* biofilm infection in cystic fibrosis. J Cyst Fibros. 2018;17:143–152. 29033275 10.1016/j.jcf.2017.08.012

