# Supplementary material for: Host-Defense-Peptide-Mimicking β-Peptide Polymer Acting as a Dual-Modal Antibacterial Agent by Interfering Quorum Sensing and Killing Individual Bacteria Simultaneously
Source: Research (Wash D C). 2023 Mar 14;6:0051. doi: 10.34133/research.0051 (PMC10014070; doi:10.34133/research.0051)
Supplement: Supplementary 1 — Fig. S1. Antimicrobial activities and stability of the 20:80-Bu:DM. Fig. S2. Cytotoxicity of the 20:80-Bu:DM in vitro. Fig. S3. PAO1 treated with 20:80-Bu:DM stained with Syto 9/PI double staining kit, showing 93% cell death compared with the untreated group. Fig. S4. 1H NMR spectrum of dye initiator in CDCl3, 600 MHz. Fig. S5. 13C NMR spectrum of dye initiator in CDCl3, 151 MHz. Fig. S6. HREI-MS spectrum of dye initiator. Fig. S7. 1H NMR spectrum of dye-poly-β-peptide, dye-20:80-Bu:DM. Fig. S8. Visualize the process of bacterial membrane damage. Fig. S9. TEM images of the PAO1 treated with PBS and 20:80-Bu:DM respectively. Fig. S10. Volcano plot of gene expression differences between P. aeruginosa biofilms and biofilms treated with 20:80-Bu:DM. Fig. S11. DEG distribution in QS-related pathway. Fig. S12. Gram staining of the lung sections, indicating the tissue distribution and the bacterial cluster size in the lungs of mice with different treatments. Fig. S13. TEM images of the PAO1 in the lung, showing that P. aeruginosa in vivo is disrupted by 20:80-Bu:DM. Fig. S14. IL-10 expression level in the lung of mice with PAO1 infection. Fig. S15. Overview images showed expression level and distribution of the secreted inflammatory factors (IL-1β, IL-6, and TNF-α) in the lungs of mice with different treatments, and the inflammation in the 20:80-Bu:DM group showed significant reduction. Fig. S16. Fig. S17. Fig. S18. 1H NMR spectrum of compound 1 in CDCl3, 400 MHz. Fig. S19. 13C NMR spectrum of compound 1 in CDCl3, 100 MHz. Fig. S20. HREI-MS spectrum of compound 1. [file research.0051.f1.docx]

**Supporting Information**

**Host Defense Peptide Mimicking β-Peptide Polymer Acting as a Dual-Modal Antibacterial Agent by Interfering Quorum Sensing and Killing Individual Bacteria Simultaneously**

Wanlin Li^1,3,#^, Ximian Xiao^2,#^, Yuchen Qi^4^, Xiuhui Lin^5^, Huiqun Hu^5^, Minqi Shi^4^, Min Zhou^2^, Weinan Jiang^2^, Longqiang Liu^2^, Kang Chen^2^, Kai Wang^3^, Runhui Liu^2^* & Min Zhou^1,4,6^*

1 Eye Center, The Second Affiliated Hospital, Zhejiang University School of Medicine, Hangzhou 310058, China.

2 State Key Laboratory of Bioreactor Engineering, Key Laboratory for Ultrafine Materials of Ministry of Education, Frontiers Science Center for Materiobiology and Dynamic Chemistry, Research Center for Biomedical Materials of Ministry of Education, School of Materials Science and Engineering, East China University of Science and Technology, Shanghai 200237, China.

3 Department of Respiratory and Critical Care Medicine, the Fourth Affiliated Hospital, Zhejiang University School of Medicine, Yiwu 223300, China.

4 Institute of Translational Medicine, Zhejiang University, Hangzhou 310029, China.

4 Department of Infectious Diseases, The Second Affiliated Hospital, Zhejiang University School of Medicine; Hangzhou, 310058, China

6 State Key Laboratory of Modern Optical Instrumentations, Zhejiang University, Hangzhou 310058, China.

^#^ These authors contributed equally: Wanlin Li and Ximian Xiao.

* Corresponding Author:

E-mail: zhoum@zju.edu.cn (Min Zhou); rliu@ecust.edu.cn (Runhui Liu)

**Materials and instrumentation**

All reagents were purchased from Sigma-Aldrich. 1-Hexene was purchased from Aladdin. Synthesized intermediates were purified using a SepaBean machine equipped with Sepaflash columns produced by Santai Technologies Inc. in China. Nuclear magnetic resonance (NMR) spectra were collected on a Bruker spectrometer at 400 MHz or 600 MHz. Gel permeation chromatography (GPC) was performed on a Waters GPC instrument equipped with a Waters 1515 isocratic HPLC pump and a Waters 2414 refractive index detector using DMF supplemented with 0.01 M LiBr as the mobile phase at a flow rate of 1 mL/min at 50 °C. The GPC was equipped by a Tosoh TSKgel Alpha-2500 column (particle size 7 µm), a Tosoh TSKgel Alpha-3000 column (particle size 7 µm) and a Tosoh TSKgel Alpha-4000 column (particle size 10 µm) linked in series. Relative number-average molecular weight (*M*_n_) and dispersion index (*Đ*) were calculated from the calibration curve with polymethylmethacrylate (PMMA) as the standard. Fourier transform infrared (FTIR) spectra were recorded on a Thermo Electron Nicolet 6700 FTIR spectrophotometer using a KBr disk. Matrix-assisted laser desorption ionization-time of flight (MALDI-TOF) mass spectra was collected using an AB SCIEX 4800plus MALDI-TOF analyzer in reflection mode equipped with a nitrogen laser emitting at 337 nm using 2,5-dihydroxybenzoic acid (DHB) as matrix. High resolution electron ionization time of flight mass spectrometry (HREI-MS) was collected on a Waters GCT.

**Method**

**Synthesis of β-lactam monomers.**

β-lactam monomers DMβ(±) and Buβ(±) were prepared by following the procedure in literature [1,2].

**Synthesis of dye-initiator.**

The dye-initiator was synthesized by following the procedure in literature with modifications [59]. A mixture of 4-morpholin-1, 8-naphthalic anhydride (2.00 g, 7.07 mmol), β-alanine ethyl ester hydrochloride (1.09 g, 7.07 mmol), and Et_3_N (2 mL, 14.13 mmol) in EtOH (70 mL) was refluxed under nitrogen atmosphere for overnight, TLC (ethyl acetate (EA) / petroleum ether (PE) = 1:1) indicated the complete consumption of the starting materials. The reaction mixture was concentrated in vacuo to obtain crude product, which was recrystallized with EA / PE to obtain compound 1 (2.20 g, 82%) as a yellow solid. ^1^H NMR (400 MHz, CDCl_3_) δ 8.58 (dd, *J* = 7.2, 1.2 Hz, 1H), 8.52 (d, *J* = 8.0 Hz, 1H), 8.42 (dd, *J* = 8.4, 1.2 Hz, 1H), 7.70 (dd, *J* = 8.8, 7.6 Hz, 1H), 7.22 (d, *J* = 8.0 Hz, 1H), 4.47 (t, *J* = 7.2 Hz, 2H), 4.14 (q, *J* = 7.2 Hz, 2H), 4.03-4.01 (m, 4H), 3.27-3.25 (m, 4H), 2.74 (t, *J* = 7.2 Hz, 2H), 1.22 (t, *J* = 7.2 Hz, 3H) (**Supplementraty Fig. 19**). ^13^C NMR (100 MHz, CDCl_3_) δ 171.49, 164.35, 163.86, 155.87, 132.76, 131.41, 130.33, 130.05, 126.29, 125.99, 123.30, 117.12, 115.11, 67.09, 60.75, 53.58, 36.16, 32.95, 14.28 (**Supplementraty Fig. 20**). HREI-MS: m/z calculated for C_21_H_22_N_2_O_5_ [M]^+^: 382.1529, found 382.1532 (**Supplementraty Fig. 21**).

A mixture of compound 1 (2.20 g, 5.75 mmol), NaOH (0.69 g, 17.26 mol) in H_2_O/THF/MeOH (30 mL/30 mL/30 mL) was stirred at room temperature for overnight. The reaction mixture was concentrated in vacuo. The solution was acidified with 4 M hydrochloric acid to pH = 2-3. The precipitate was collected by filtration to afford compound 2 as a yellowish solid. Without further purification, compound 2 was dried under vaccum followed by addition of N-Hydroxysuccinimide (NHS) (0.66 g, 5.75 mmol) and 1-(3-dimethylaminopropyl)-3-ethylcarbodiimide hydrochloride (EDC.HCI) (1.65 g, 8.63 mmol) and anhydrous THF (5 mL) at ice bath. Then, the solution was stirred for 24 h at room temperature and evaporated under vacuum to dryness. The resulting crude residue was dissolved in CH_2_Cl_2_ (80 mL). The organic phase was washed with DI water (2 × 100 mL) and brine (1 × 100 mL). The organic solvent was dried over anhydrous MgSO_4_ and removed under vacuum to give crude product. The crude product was recrystallized with CH_2_Cl_2_ / PE to obtain the desired dye-initiator (0.93 g, 36%). ^1^H NMR (600 MHz, CDCl_3_) δ 8.61 (dd, *J* = 7.2, 1.2 Hz, 1H), 8.55 (d, *J* = 8.4 Hz, 1H), 8.44 (dd, *J* = 8.4, 1.2 Hz, 1H), 7.72 (dd, *J* = 8.4, 7.2 Hz, 1H), 7.24 (d, *J* = 7.8 Hz, 1H), 4.58 (t, *J* = 7.2 Hz, 2H), 4.03-4.01 (m, 4H), 3.28-3.27 (m, 4H), 3.12 (t, *J* = 7.8 Hz, 2H), 2.82 (s, 4H). ^13^C NMR (150 MHz, CDCl_3_) δ 168.96, 166.60, 164.31, 163.80, 156.07, 133.02, 131.64, 130.55, 130.14, 126.31, 126.04, 123.15, 116.91, 115.16, 67.11, 53.59, 35.30, 29.68, 25.71. HREI-MS: m/z calculated for C_23_H_21_N_3_O_7_ [M]^+^: 451.1380, found 451.1375.

**Synthesis and characterizations of 20:80-Bu:DM**

To synthesize β-peptide polymer 20:80-Bu:DM, a polymerization was performed in a glovebox under nitrogen atmosphere at room temperature (rt). Two β-lactam monomers DM (50 mg, 0.22 mmol) and Bu (5.6 mg, 0.055 mmol) were dissolved and mixed in anhydrous tetrahydrofuran (THF) (0.68 mL). Then, adding co-initiator *t*BuBzCl in anhydrous THF (0.07 mL, 0.014 mmol) and the catalyst Lithium bis(trimethylsilyl)amide (LiHMDS) in anhydrous THF (0.14 mL, 0.055 mmol) into the reaction mixture sequentially, the mixture was stirred for 6 hours. Once the monomers were consumed completely, the reaction mixture was quenched with MeOH. The NHBoc protected polymer was obtained by repeated dissolution-precipitation-centrifugation using THF and PE for three times. The NHBoc protected polymer was characterized by gel permeation chromatography (GPC) using *N*, *N*-dimethylformamide (DMF) as the mobile phase.

NHBoc protecting groups were removed by dissolving the polymer in TFA at room temperature under shaking for 2 hours. Then, TFA was removed by air flow to obtain a viscous liquid. The crude product was purified by repeated dissolution-precipitation-centrifugation using MeOH and methyl tert-butyl ether for three times. The purified polymer was dissolved in milli-Q water and filtered with polytetrafluoroethylene filter (0.45 μm) and lyophilized to give a white powder. The polymer was characterized by ^1^H NMR spectra using D_2_O as the deuterated solvent.

**Synthesis and characterization of dye-20:80-Bu:DM**

To synthesize dye-peptide polymer 20:80-Bu:DM, a polymerization was performed in a glovebox under nitrogen atmosphere at room temperature. Two β-lactam monomers DM (37.3 mg, 0.16 mmol) and Bu (5.2 mg, 0.04 mmol) were dissolved and mixed in anhydrous THF (0.56 mL). Then, adding co-initiator dye-initiator in anhydrous THF (0.055 mL, 0.01 mmol) and the catalyst Lithium bis(trimethylsilyl)amide (LiHMDS) in anhydrous THF (0.11 mL, 0.04 mmol) into the reaction mixture sequentially, the mixture was stirred for 6 hours. Once the monomers were consumed completely, dye-peptide polymer 20:80-Bu:DM was obtained by following aforementioned purification and deprotection operations. The polymer was characterized by ^1^H NMR spectra using D_2_O as the deuterated solvent.

***In vitro* biocompatibility of 20:80-Bu:DM**

For cytotoxicity comparison between the 20:80-Bu:DM and magainin II, NIH 3T3 fibroblast cells (ATCC CRL-1658) were seeded in 96-well plates at a density of 10^4^ cells per well and incubated overnight in Dulbecco’s modified Eagle’s medium (DMEM). After the old medium was removed, 100 µL solution (20:80-Bu:DM and magainin II) in DMEM in a two-fold serial dilution series were added into each well at concentrations ranging from 6.25 to 200 µg/mL for 24 h incubation. The cell viability was measured with the MTT assay kit.

The cytotoxicity of 20:80-Bu:DM was also evaluated with the human bronchial epithelial cells Beas-2B, and lung adenocarcinoma cells A549. For the Cell Counting Kit-8 (CCK-8) assay and, cells were seeded in 96-well plates (5000 per well) and cultured with DMEM (10% FBS, 1% Penicillin-Streptomycin Solution) till 50% confluency. After the old medium was removed, 100 µL 20:80-Bu:DM and magainin II solution in DMEM in a two-fold serial dilution series were added into each well at concentrations ranging from 6.25 to 50 µg/mL for 24 h of incubation. Discard the medium and add CCK-8. The cell viability was measured with the SpectraMax M5 Multi-Mode Microplate Reader (Molecular Devices, America) at 450 nm.

For the living/dead cell double staining method, the adherent cells were incubated with 25 μg/mL of 20:80-Bu:DM at 37 °C for 24 h. The treated cells were stained with the Calcein-AM/PI Double Stain Kit (YEASEN, Shanghai, China) and observed by a fluorescence microscope.

**RNA-seq**

PAO1 was statically cultured for 24 h to form the biofilm in the tube. After treated with PBS or 20:80-Bu:DM (50 μg/mL) for 6 hours, the suspensions were centrifugated for bacterial collection. The collected bacteria were quick-frozen with the liquid nitrogen and subsequently sent to BGI-Shenzhen for RNA-seq analysis. The RNA-seq was performed as the following: (1) Extract bacteria total RNA using the standard method. (2) By using Agilent 2100 Bioanalyzer (Agilent RNA 6000 Nano Kit), the total RNA samples’ concentration, RIN, 28S/18S, and size were detected. (3) DNase I degraded double-stranded and single-stranded DNA presenting in RNA samples. (4) Total RNA sample was treated with Ribo-Zero™ Magnetic Gold Kit /Plant Kit(epicenter) to deplete rRNA. (5) RNA molecules were fragmented into small pieces using fragmentation reagent. (6) First-strand cDNA was generated using random primers reverse transcription, followed by a second-strand cDNA synthesis. (7) The synthesized cDNA was subjected to end-repair and then was 3’ adenylated. Adapters were ligated to the ends of these 3’ adenylated cDNA fragments. (8) This process was to amplify the cDNA fragments with adaptors from previous step. PCR products were purified with the XP beads, and dissolved in EB solution. (9) The libraries were assessed quality and quantity in two methods: check the distribution of the fragments size using the Agilent 2100 bioanalyzer, and quantify the library using real-time quantitative PCR (QPCR) (TaqMan Probe). (10) The qualified libraries were amplified on cBot to generate the cluster on the flowcell. And the amplified flowcell will be sequenced pair end on the Illumina System.


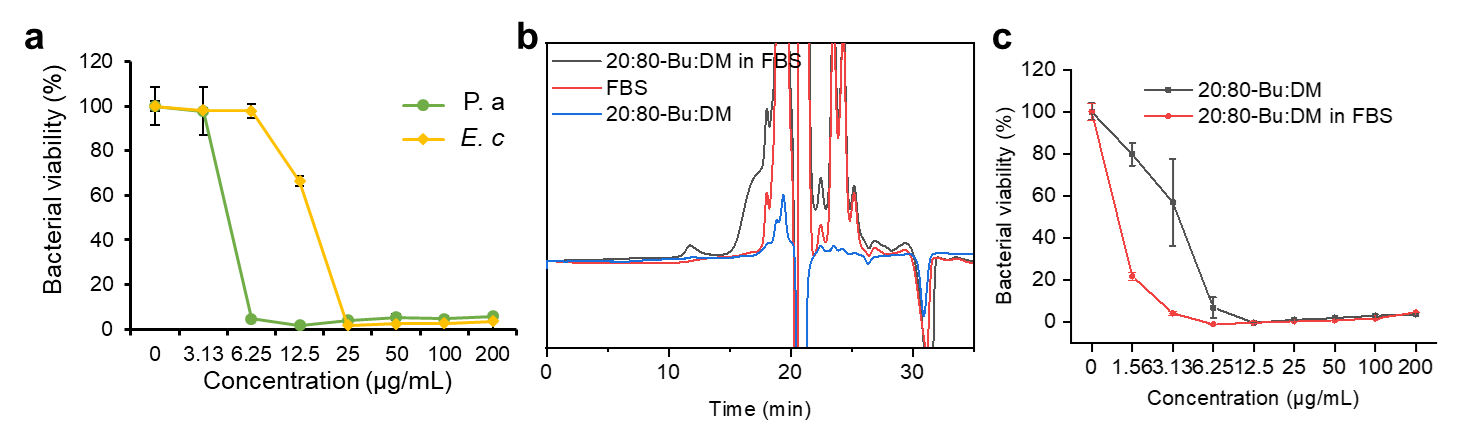


**Supplementary Figure 1.** Antimicrobial activities and stability of the 20:80-Bu:DM. a) The MIC of20:80-Bu:DM against *E. coli* and *P. aeruginosa*, the MIC for *E. coli* is 25 μg/mL, and for PAO1 is 6.25 μg/mL. b) The water GPC characterization of 20:80-Bu:DM treated with FBS for 1 week or not. c) The MIC of 20:80-Bu:DM against *P. aeruginosa* treated with FBS for 1 week or not.


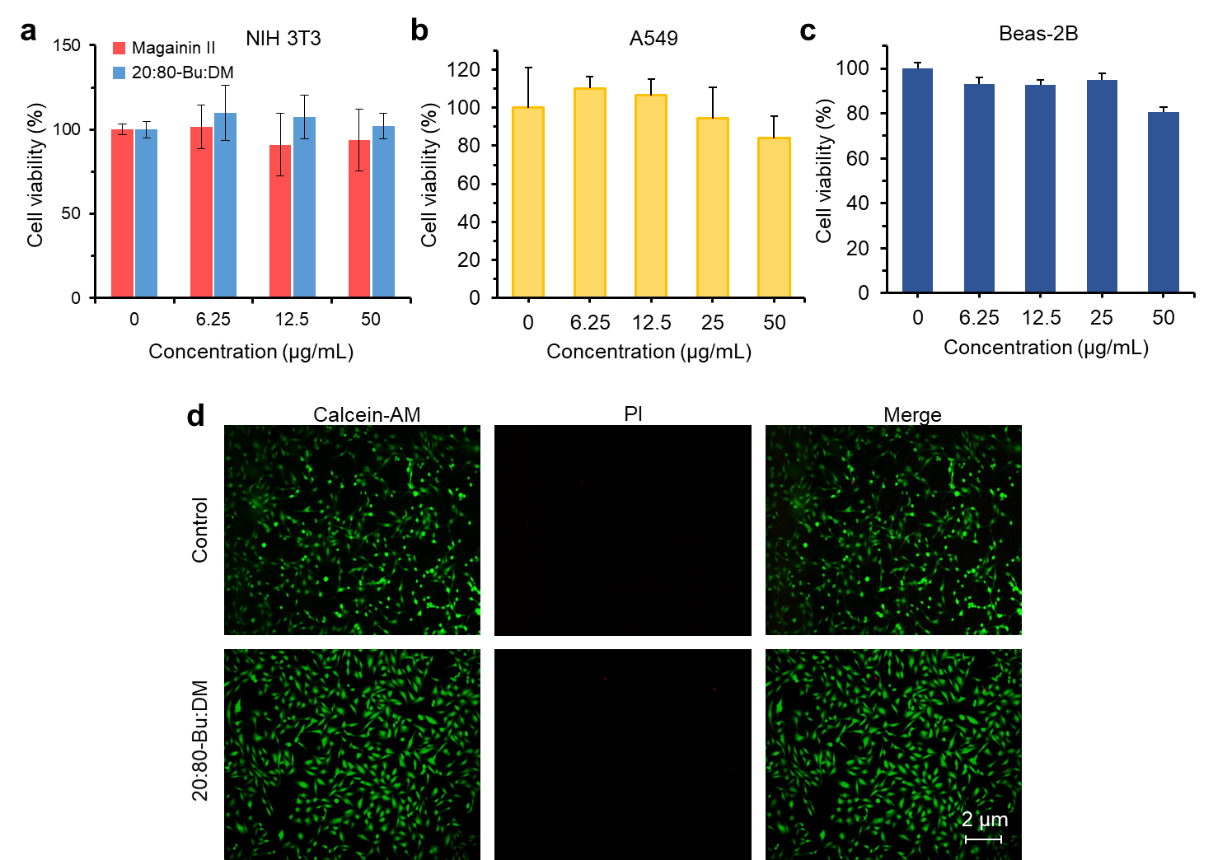


**Supplementary Figure 2.** **Cytotoxicity of the 20:80-Bu:DM *in vitro*.** a) Comparison of the *in vitro* cytotoxicity of the 20:80-Bu:DM with magainin II to the NIH3T3 cells (*n* = 5). b) *In vitro* cytotoxicity of the 20:80-Bu:DM to the A549 cells (*n* = 5). c) *In vitro* cytotoxicity of the 20:80-Bu:DM to the Beas-2B cells (*n* = 5). d) Live/dead double-stained Beas-2B cells which have incubated with 20:80-Bu:DM (25 μg/mL) for 24 h, living cells in green and dead cells in red, showing the biocompatibility of the 20:80-Bu:DM *in vitro* (*n* = 5).


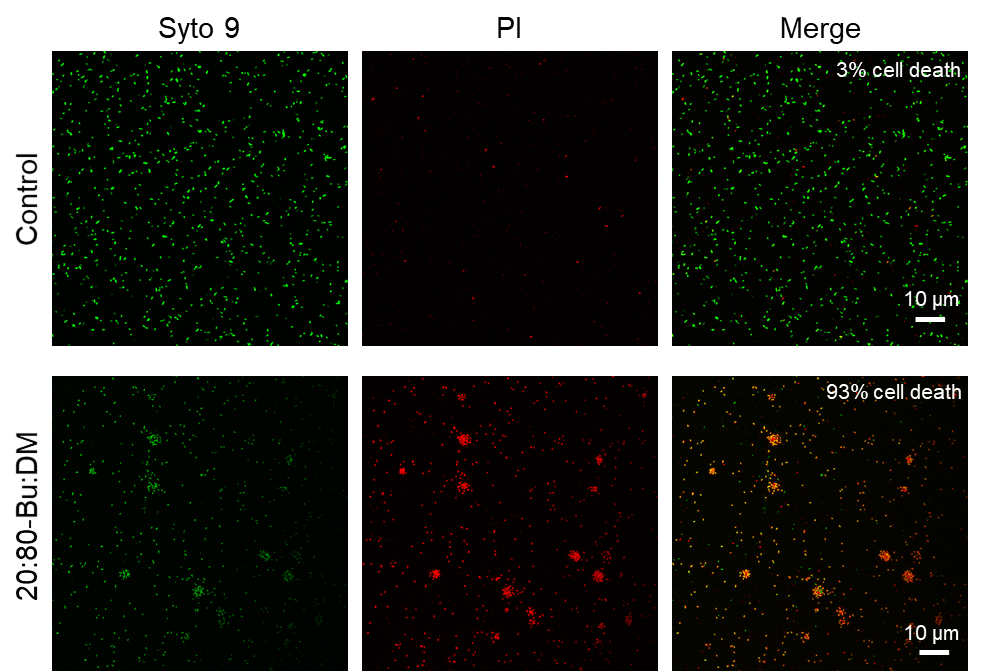


**Supplementary Figure 3** PAO1 treated with 20:80-Bu:DM stained with Syto 9/PI double staining kit, showing 93% cell death compared with the untreated group.


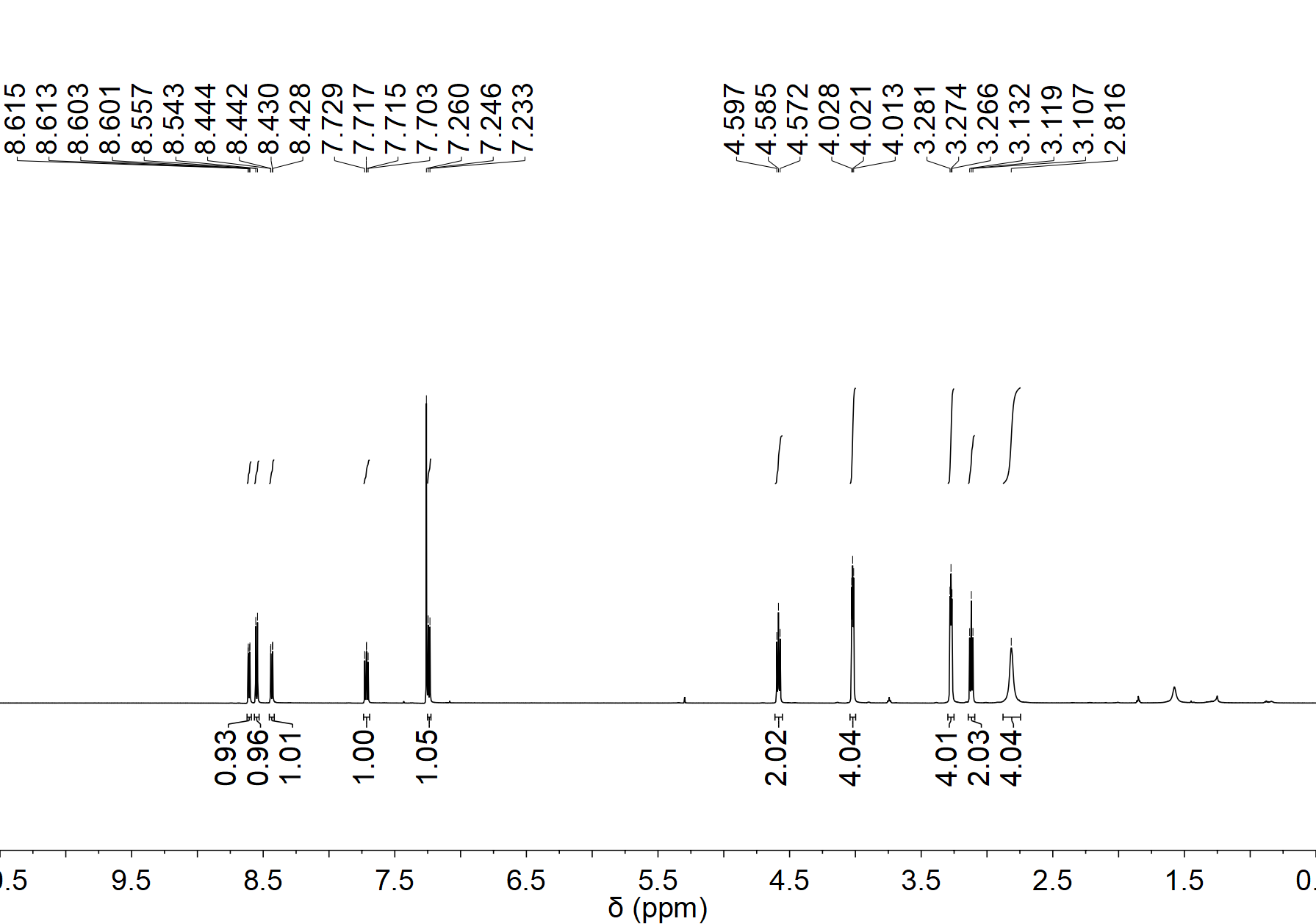


**Supplementary Figure 4**. ^1^H NMR spectrum of dye-initiator in CDCl_3_, 600 MHz.


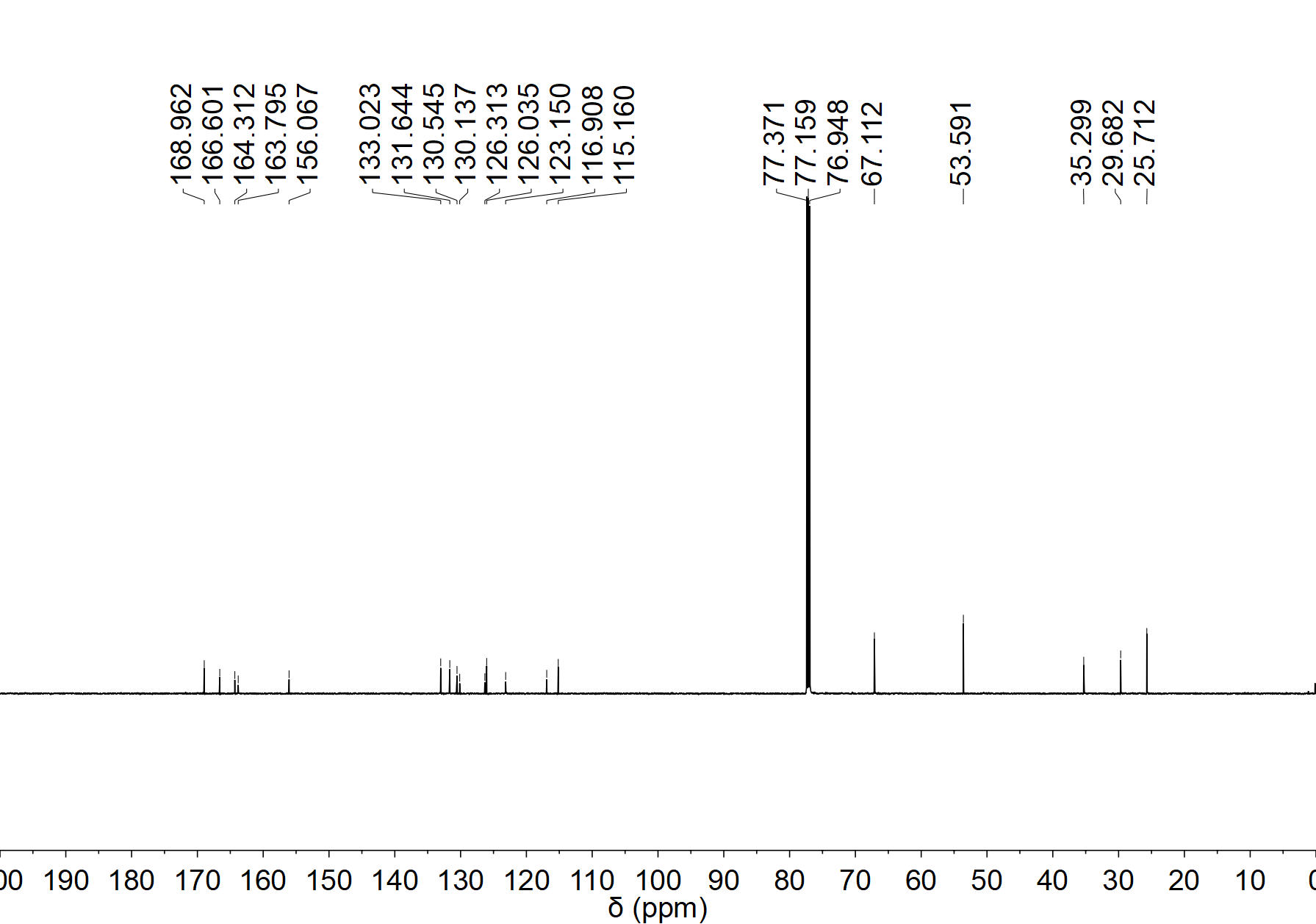


**Supplementary Figure 5**. ^13^C NMR spectrum of dye-initiator in CDCl_3_, 151 MHz.


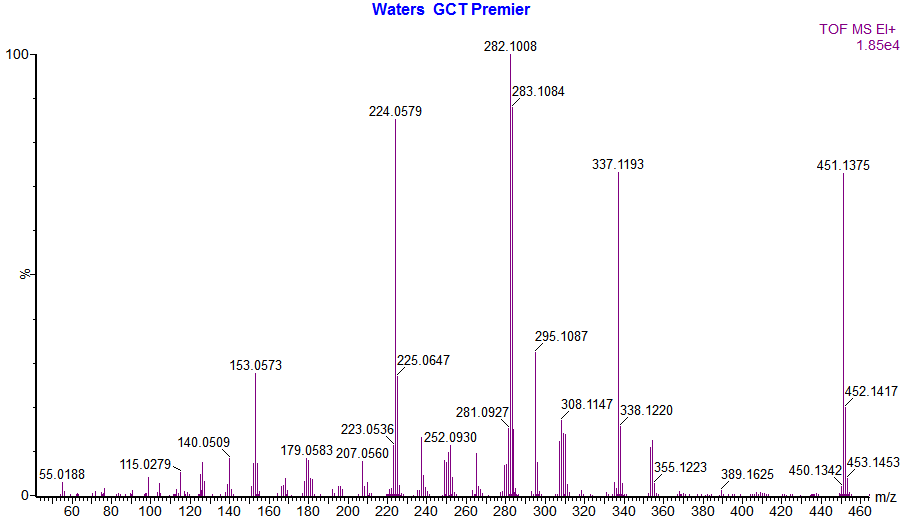


**Supplementary Figure 6**. HREI-MS spectrum of dye-initiator.


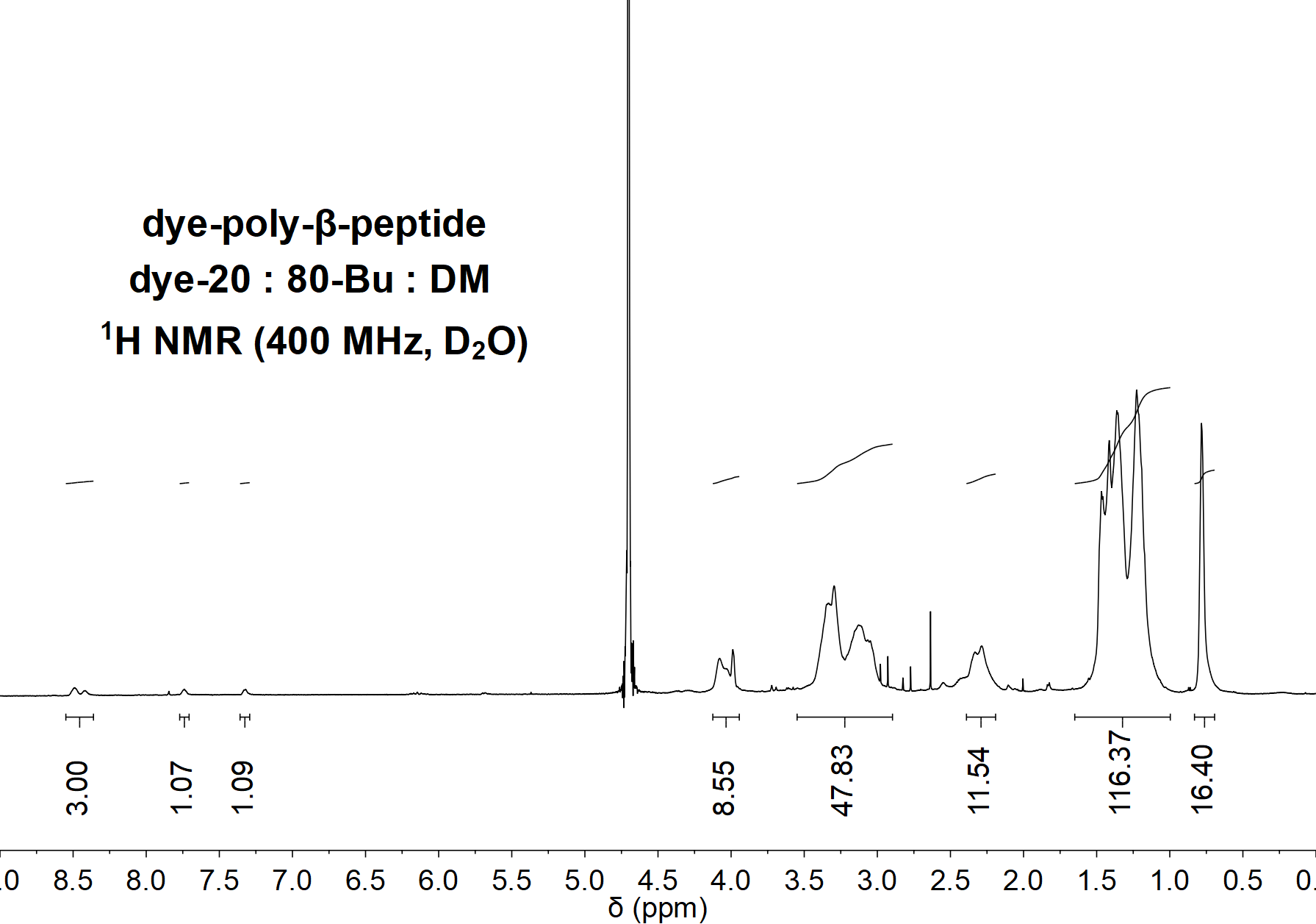


**Supplementary Figure 7**. ^1^H NMR spectrum of dye-poly-β-peptide, dye-20:80-Bu:DM.


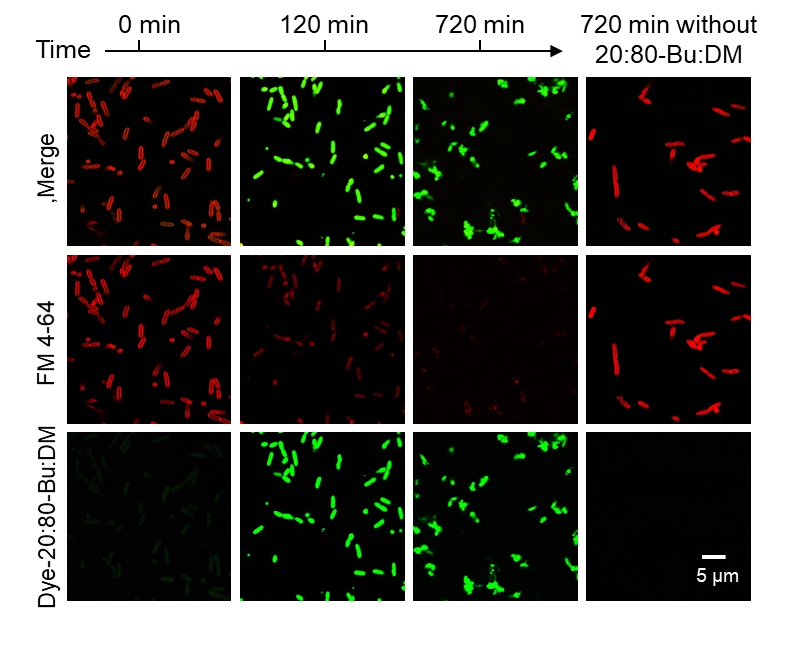


**Supplementary Figure 8.** Visualize the process of bacterial membrane damage. Bacterial membranes were stained with FM 4-64 and showed as red lines, dye-20:80-Bu:DM inserted into the membranes showed green in the images, as the incubation time increased, the red fluorescence gradually disappeared. 720 min incubation of the bacteria without dye-20:80-Bu:DM proved that the fluorescence of FM 4-64 will not be quenched naturally with time increasing in 720 min.


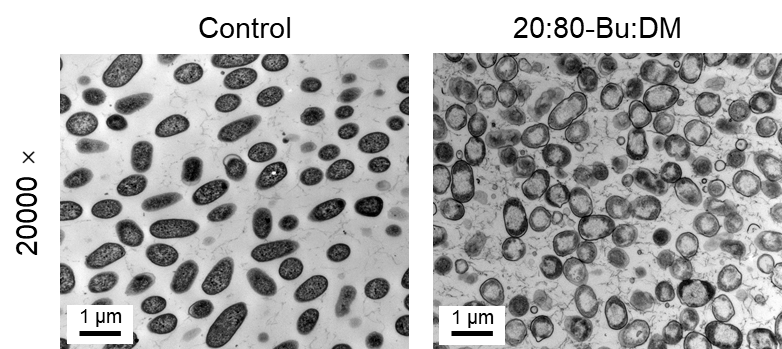


**Supplementary Figure 9.** TEM images of the PAO1 treated with PBS and 20:80-Bu:DM respectively.


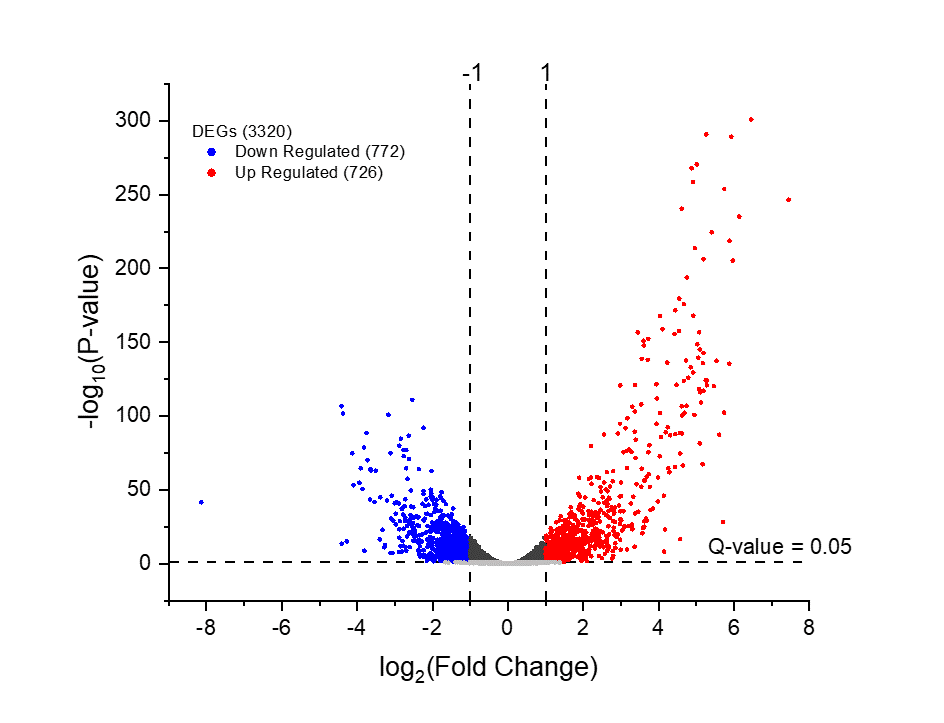


**Supplementary Figure 10.** volcano plot of gene expression differences between *P. aeruginosa* biofilms and biofilms treated with 20:80-Bu:DM.


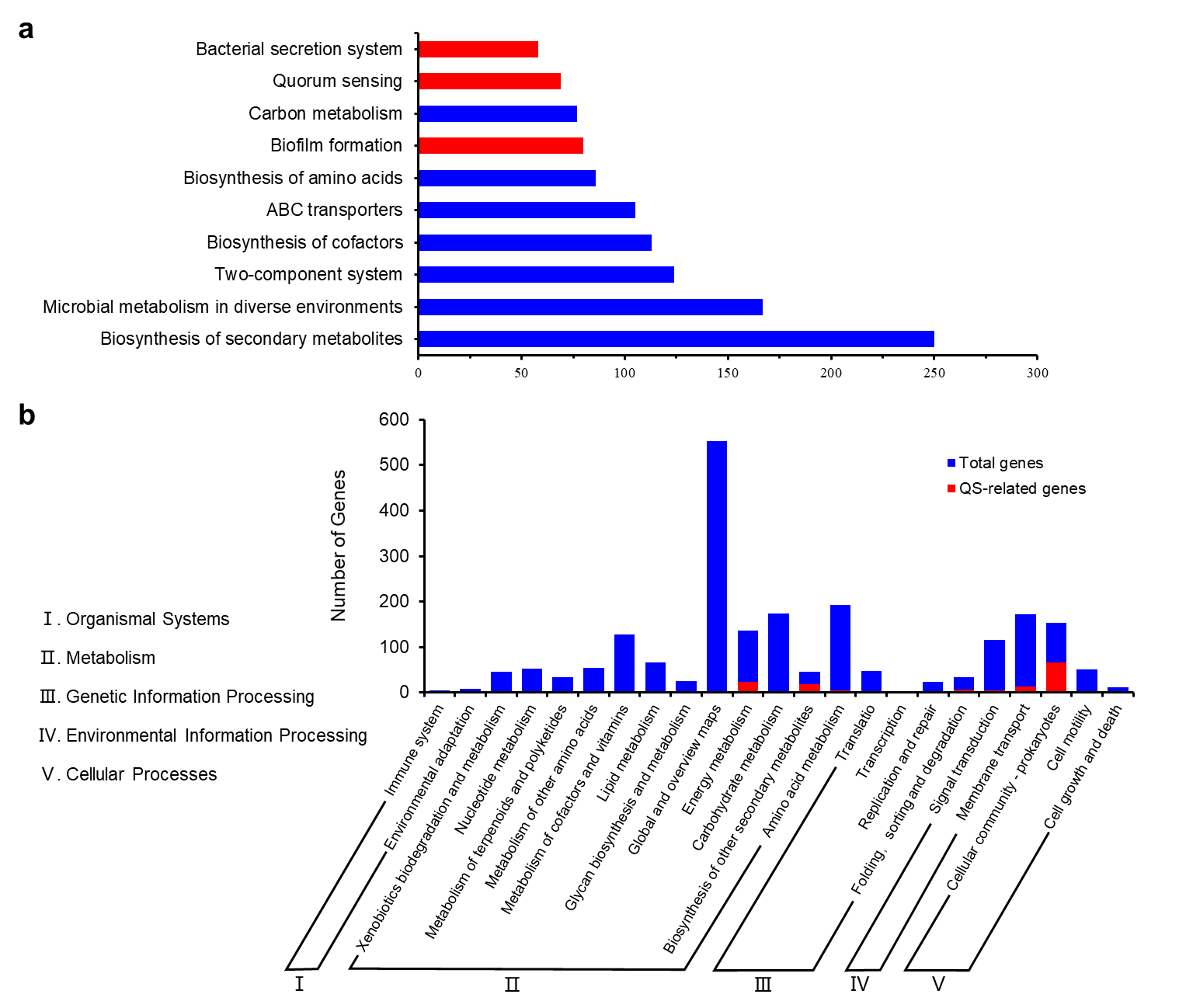


**Supplementary Figure 11.** **DEGs distribution in QS-related pathway. a** The top 10 KEGG pathways statistically have the most DEGs, including quorum sensing and biofilm formation pathways. **b** KEGG pathway classification map, the map classified DEGs located pathways into five items according to the corresponding functions, and the distribution of QS-related genes was marked in red, indicating that 20:80-Bu:DM mainly influenced the metabolism function of PAO1.


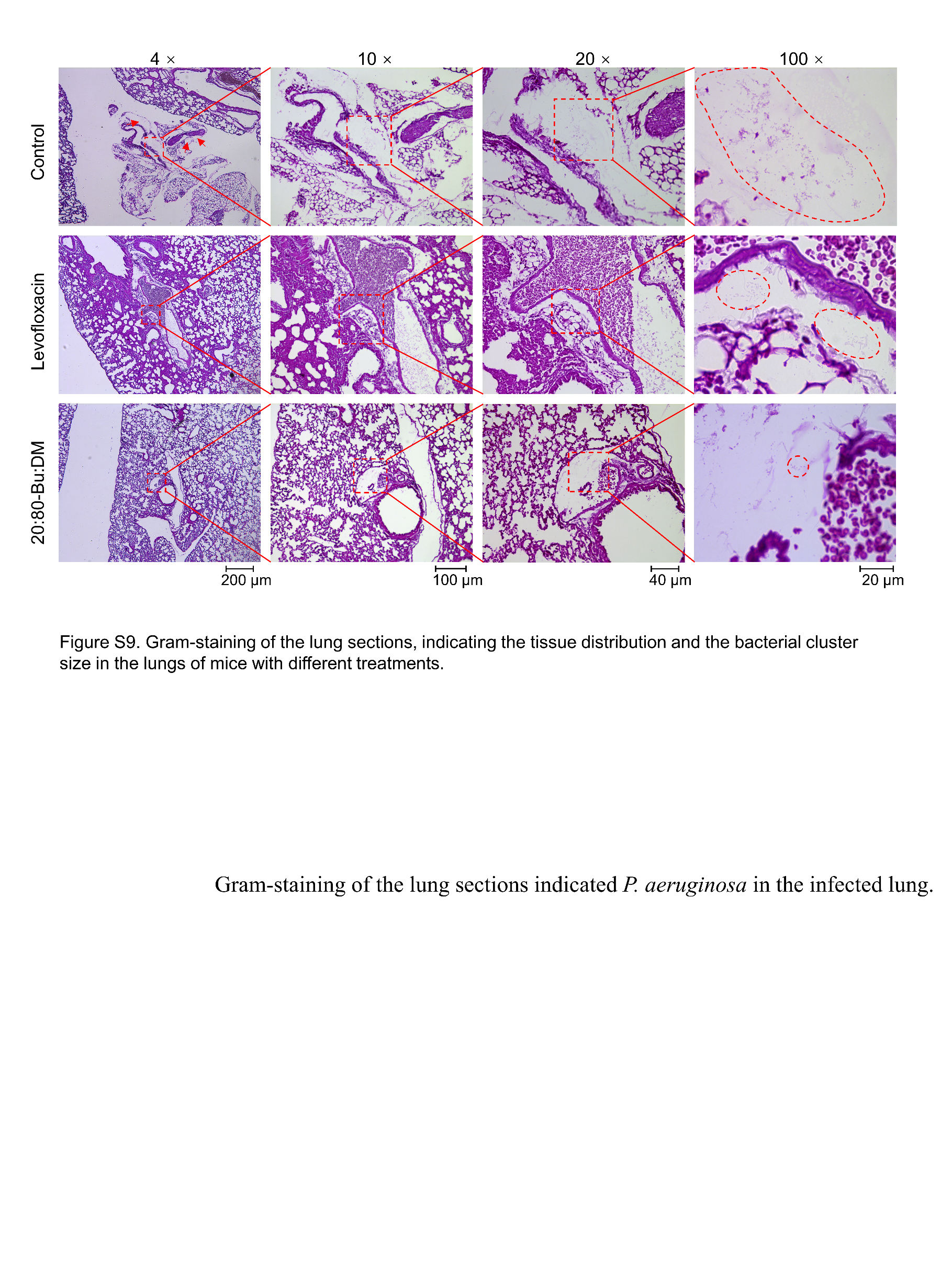


**Supplementary Figure 12.** Gram-staining of the lung sections, indicating the tissue distribution and the bacterial cluster size in the lungs of mice with different treatments.


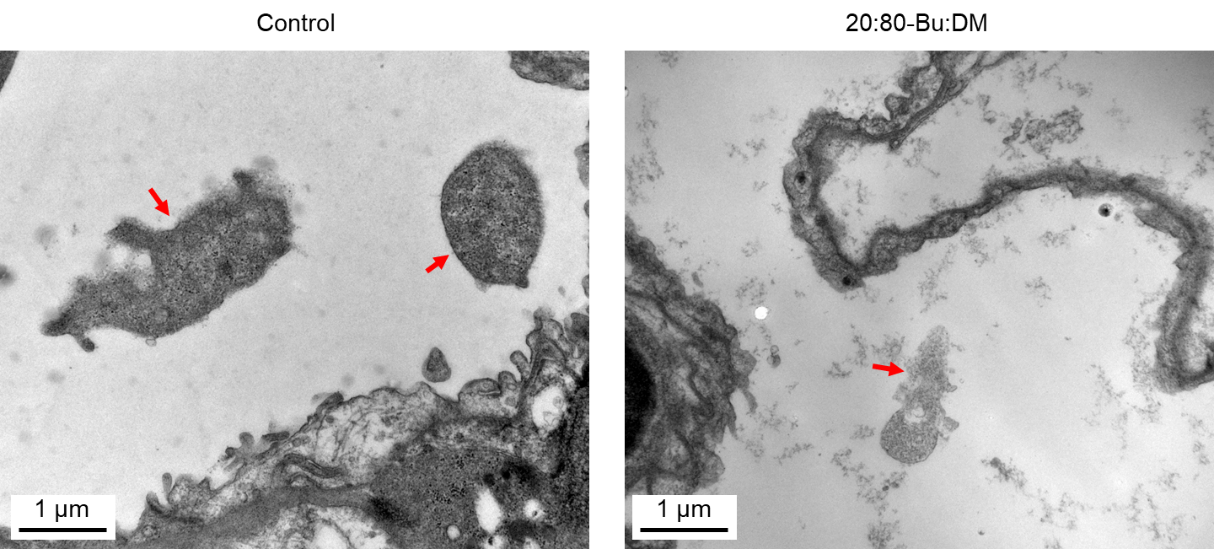


**Supplementary Figure 13.** TEM images of the PAO1 in the lung, showing that *P. aeruginosa in vivo* disrupted by 20:80-Bu:DM.


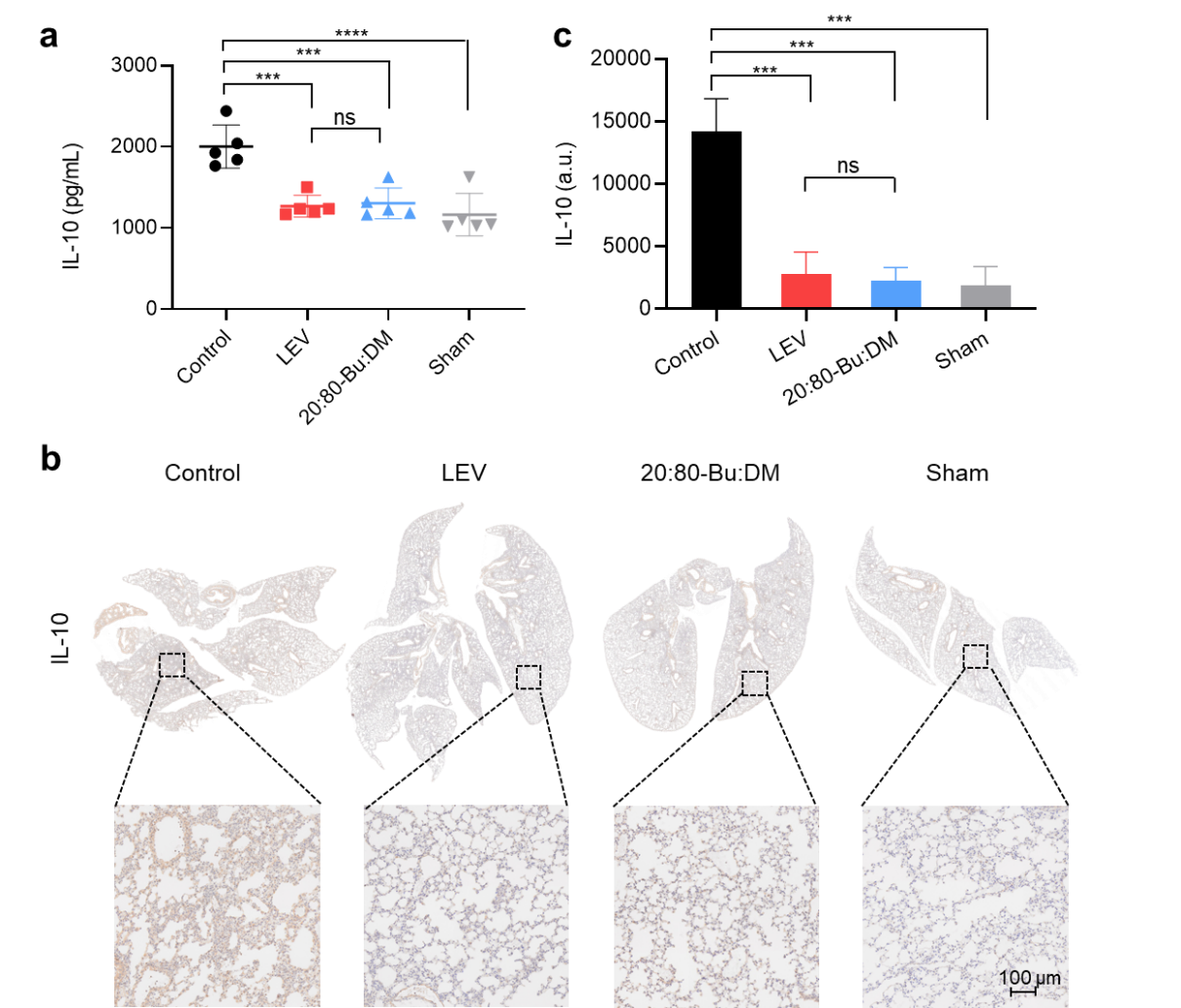


**Supplementary Figure 14. IL-10 expression level in the lung of mice with PAO1 infection. a** The IL-10 detection in lung homogenates of PAO1 infection mice using the Elisa method, the IL-10 expression level showed a statistically significant reduction in the 20:80-Bu:DM-treated mice but has no significant difference with the LEV administrated mice (*n* = 5). **b** Overview and the corresponding enlarged images of the immunohistochemical analysis of the IL-10 expression and distribution in the lung with PAO1 infection, and the IL-10 quantification was shown in **c** (*n* = 5). Significance of differences was determined using the one-way ANOVA method. *p<0.05, **p < 0.01, ***p < 0.001, ****p <0.0001.


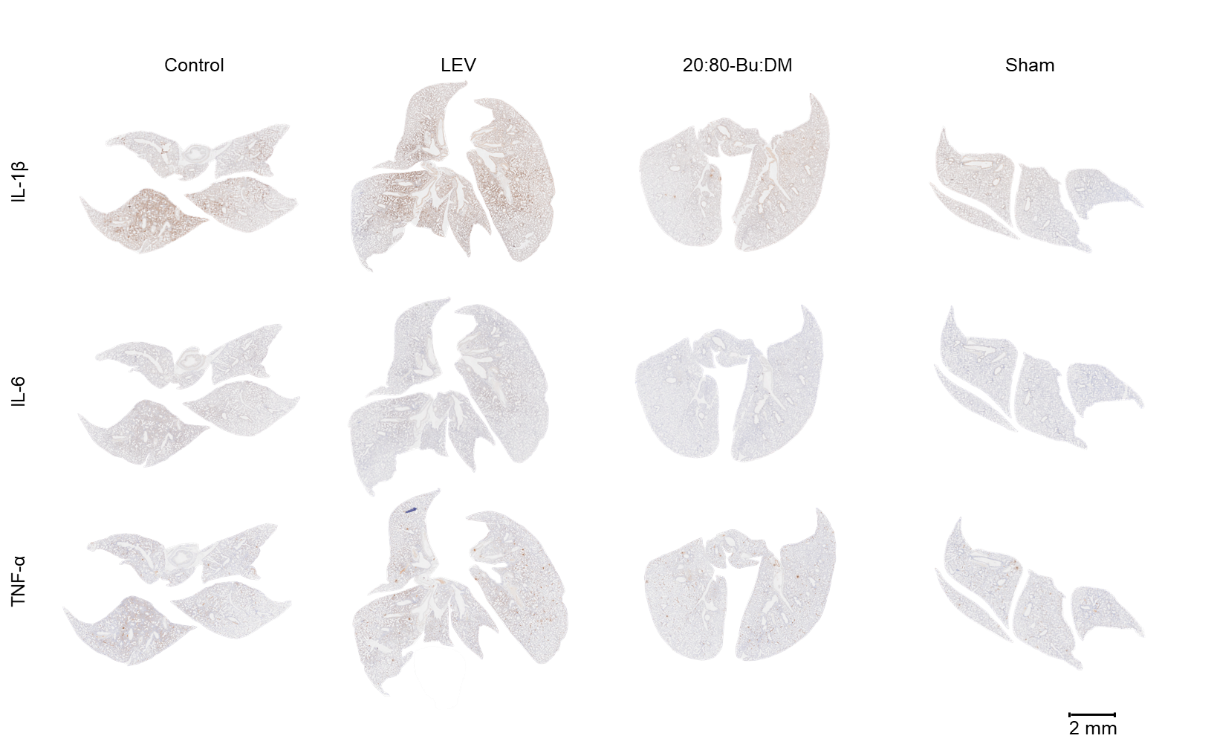


**Supplementary Figure 15.** Overview images showed expression level and distribution of the secreted inflammatory factors (IL-1β, IL-6, and TNF-α) in the lungs of mice with different treatments, and the inflammation in the 20:80-Bu:DM group showed significant reduction.


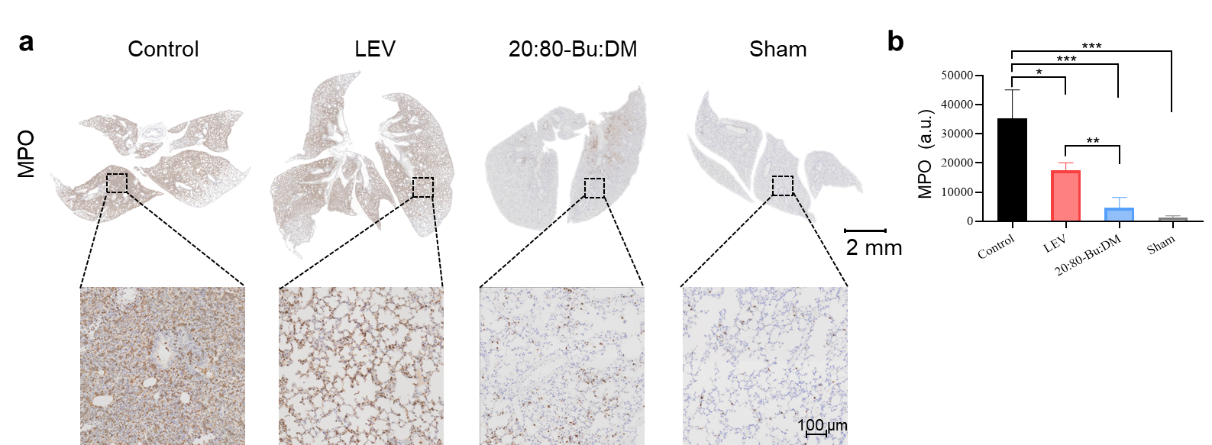


**Supplementary Figure 16**. **a** Overview and local images of the immunohistochemical analysis of the MPO secretion in lungs of mice with different treatments, representing the neutrophils infiltration in the tissue. **b** Quantitative analysis of the MPO expression level in the lungs, and the values showed significantly reduced in the 20:80-Bu:DM group compared with the control and LEV groups, suggesting less neutrophil infiltration (*n* = 5). Significance of differences was determined using the one-way ANOVA method. *p<0.05, **p < 0.01, ***p < 0.001, ****p <0.0001.


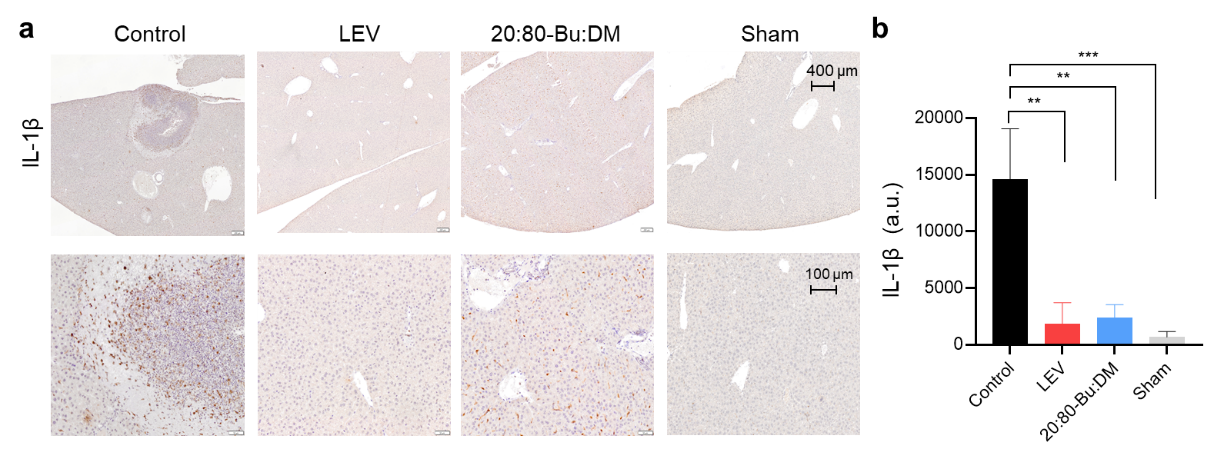


**Supplementary Figure 17.** **a** Images of the IL-1β expression and distribution in the liver of the ECPLA mice, measuring by immunohistochemistry. **b** Quantitative results of the above images, the IL-1β had no significant increased expression, indicating the healing infection (*n* = 5). Significance of differences was determined using the one-way ANOVA method. *p < 0.05, **p < 0.01, ***p < 0.001, ****p <0.0001.


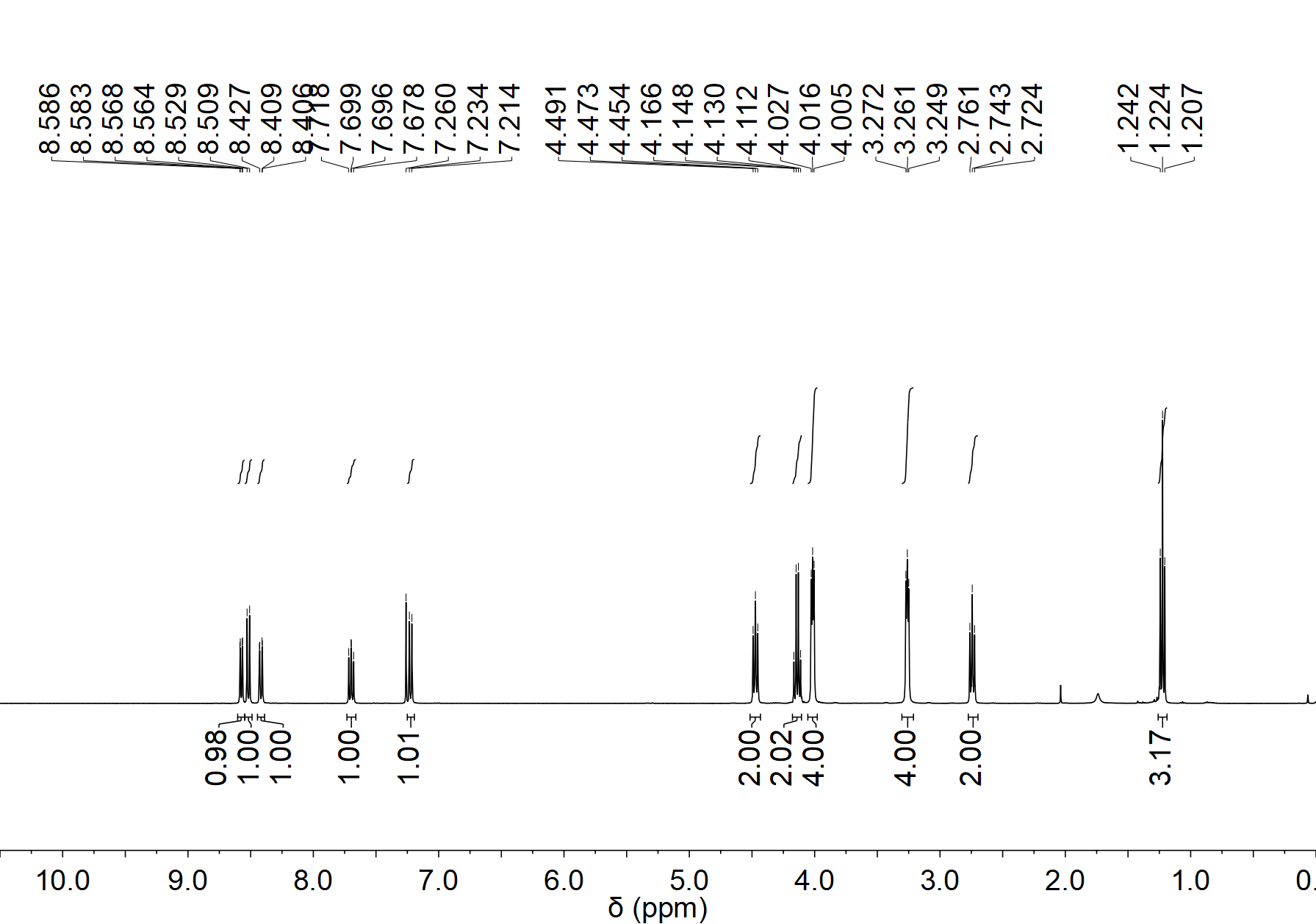


**Supplementary Figure 18.** ^1^H NMR spectrum of compound 1 in CDCl_3_, 400 MHz.


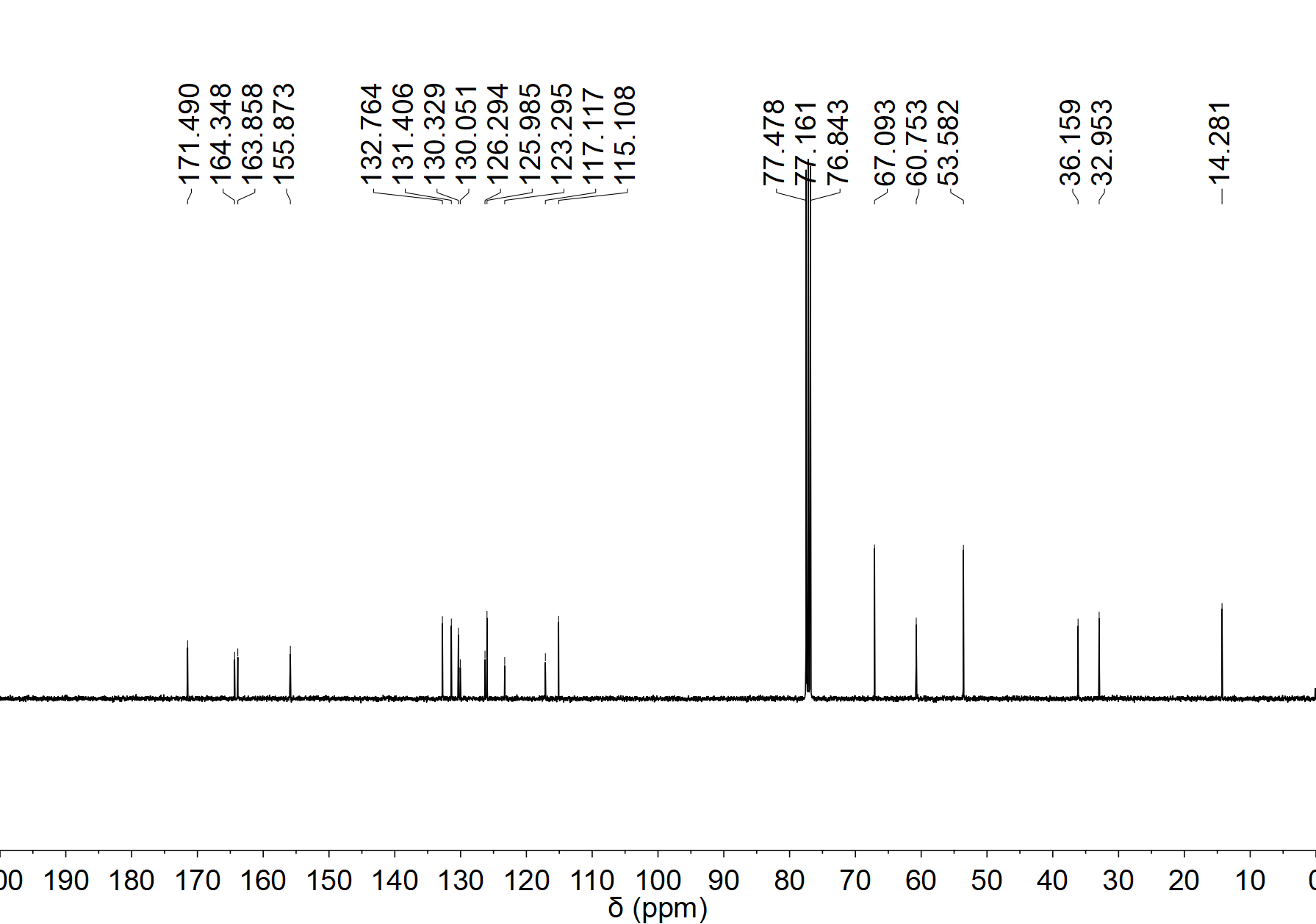


**Supplementary Figure 19**. ^13^C NMR spectrum of compound 1 in CDCl_3_, 100 MHz.


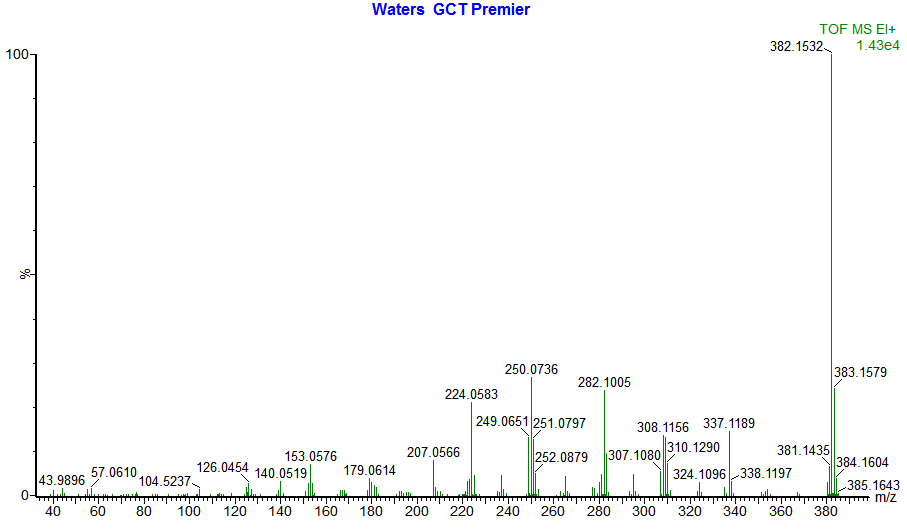


**Supplementary Figure 20**. HREI-MS spectrum of compound 1.

**Reference**

[1] J. Zhang, D.A. Kissounko, S.E. Lee, S.H. Gellman, S.S. Stahl, Access to Poly-β-Peptides with Functionalized Side Chains and End Groups via Controlled Ring-Opening Polymerization of β-Lactams, J. Am. Chem. Soc. 131 (2009) 1589–1597. https://doi.org/10.1021/ja8069192.

[2] D. Shellhamer, K. Alexander, S. Bunting, S. Elwin, C. Licata, J. Milligan, R. Robinson, D. Shipowick, L. Smith, M. Perry, Improved Synthetic Utility of a Sluggish Electrophile: Reaction of Chlorosulfonyl Isocyanate with Unreactive and Reactive Alkenes, Synthesis. 47 (2015) 1944–1950. https://doi.org/10.1055/s-0034-1380553.

[3] J. Wu, T. Yi, T. Shu, M. Yu, Z. Zhou, M. Xu, Y. Zhou, H. Zhang, J. Han, F. Li, C. Huang, Ultrasound Switch and Thermal Self-Repair of Morphology and Surface Wettability in a Cholesterol-Based Self-Assembly System, Angew. Chemie Int. Ed. 47 (2008) 1063–1067. https://doi.org/10.1002/anie.200703946.
